# Spatial Transcriptomics of TMJ Reveals a Remodeling Fibroblast‐Immune Microenvironment Driving Arthritis Pain

**DOI:** 10.1002/advs.202519816

**Published:** 2026-01-07

**Authors:** Ziying Lin, Supawadee Jariyasakulroj, Yang Shu, Jingyi Chen, Qing Chang, Pao‐Fen Ko, Yuyueyang Qiu, Feixiang Chen, David Ahn, Zhen Zhao, Jian‐Fu Chen

**Affiliations:** ^1^ Center for Craniofacial Molecular Biology University of Southern California Los Angeles California USA; ^2^ Department of Masticatory Science, Faculty of Dentistry Mahidol University Bangkok Thailand; ^3^ Department of Physiology and Neuroscience Keck School of Medicine University of Southern California Los Angeles California USA; ^4^ Oral & Maxillofacial Surgery San Francisco VA Medical Center San Francisco California USA

**Keywords:** arthritis, fibroblast‐immune interaction, pain, spatial transcriptomics, temporomandibular joint

## Abstract

Temporomandibular joint (TMJ) arthritis remodels the cartilage, subchondral bone, and synovial tissue with diverse cell changes. The functional importance of the anatomical organization of TMJ cell types and cellular microenvironment in painful arthritis remains largely unknown. Here, we applied seqFISH (sequential Fluorescence In Situ Hybridization) spatial transcriptomics to examine the adult mouse TMJ. We uncovered new cell types and comprehensively mapped anatomical locations of diverse cell types with distinct neighborhoods, revealed arthritis‐induced cell number and cell status changes, and discovered microenvironment remodeling of fibroblast‐immune cells, which are confirmed in patient synovial tissues. Functional and mechanistic studies showed that macrophage‐specific knockout of mouse *Igf1* promotes its immune activation and upregulates *Il33* in adjacent synovial fibroblasts, resulting in inflammatory fibroblast expansion. In turn, fibroblast‐specific deletion of *Il33* alleviates inflammatory macrophages and inflammation, leading to pain mitigation. Thus, spatial transcriptomics maps diverse cell types in TMJ and reveals a remodeling of synovial fibroblast‐immune microenvironment via the *Igf1‐Il33* axis, which drives arthritis pain with therapeutic potentials.

## Graphical abstract

Spatial transcriptomics reveals a remodeled fibroblast‐immune microenvironment in the temporomandibular joint (TMJ) during arthritis. By combining seqFISH with genetic mouse models, this study uncovers TMJ spatial cell atalas, macrophage‐fibroblast crosstalk, and cytokine signaling pathways driving TMJ inflammation and pain. These findings provide new cellular insights into TMJ arthritis and open avenues for targeted therapies in orofacial pain disorders.

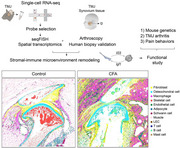

## 1 Introduction

The temporomandibular joint (TMJ) is a complex structure comprising bone, muscle, and cartilaginous tissues that collectively facilitate functions such as eating, speaking, and breathing [1, 2]. TMJ alterations could lead to temporomandibular disorders (TMDs), a group of conditions that represent the second most prevalent musculoskeletal pain disorder after chronic lower back pain [3]. Among these, arthrogenous TMD is characterized by inflammation, dysfunction, and degeneration of the hard and soft tissues within the joint [2]. TMJ arthritis is a common and debilitating subtype of arthrogenous TMD, which significantly impairs quality of life due to persistent jaw pain and restricted jaw mobility [3]. Despite its clinical significance, TMJ arthritis remains understudied compared to other synovial joints such as the knee, shoulder, and hip. This gap is partly due to the TMJ's complex anatomy as well as its distinct developmental, genetic, and molecular programs. TMJ fibrocartilage developmentally originates from the neural crest, unlike the mesoderm‐derived hyaline cartilage of limb joints [4]. The TMJ is structurally lined with fibrocartilage containing both types I and II collagen, whereas articular surfaces of limb joints consist solely of type II collagen‐rich hyaline cartilage [5]. This composition equips the TMJ to better resist tensile forces, reflecting its unique functional demands. Diverse TMJ‐resident cell types and their interactions are likely central to the pathogenesis of TMJ arthritis, yet this cellular complexity remains poorly understood.

Preclinical animal models are essential for advancing our understanding of the pathophysiology of TMJ arthritis and pain. Unlike the knee joint, human TMJ tissue is difficult to obtain, which is particularly true for healthy controls. In rare cases of TMJ whole‐joint replacement, the available tissue is typically end‐stage and of poor quality [6, 7]. Moreover, such samples reflect a mixture of primary and secondary effects, making it challenging to dissect early and disease‐driving processes. To investigate TMD pathogenesis and treatment, a wide range of preclinical models have been developed and are generally categorized into four major approaches: chemical induction, mechanical stimulation, surgical operation, and genetic modification. TMJ inflammation plays a key role in subgroups of patients, particularly in association with disorders such as osteoarthritis, rheumatoid arthritis, and gout, where inflammatory activity within the TMJ is heightened. We recently established a CFA (complete Freund's adjuvant)‐induced inflammatory arthritis TMJ mouse model that recapitulates key features of TMJ arthritis, including joint inflammation and orofacial pain behaviors [8, 9]. This model provides a valuable platform for studying the diverse TMJ cell types and their interactions, evaluating potential therapeutic strategies under controlled and reproducible conditions.

Emerging single‐cell RNA sequencing (scRNA‐seq) studies have provided valuable insights into the TMJ by identifying cell types and transcriptional programs associated with disease pathophysiology [8, 10, 11, 12, 13]. However, the tissue dissociation required for scRNA‐seq eliminates spatial context and provides a challenge to detect rare cell sub‐populations, limiting the ability to examine local cell‐cell interactions that are critical in TMJ arthritis. Spatial transcriptomics preserves the spatial organization of tissues, enabling the interrogation of cell‐type‐specific gene expression within the native tissue architecture. Although both sequencing‐based and probe‐based spatial transcriptomics platforms are available, most applications to date have focused on soft tissues [14, 15, 16]. Applying these technologies to complex joint structures containing bone and cartilage remains technically challenging but is essential for advancing spatially resolved studies of joint disease, including TMJ arthritis.

In this study, we investigated the normal and arthritic TMJ with pain at single‐cell resolution using the probe‐based spatial transcriptomics method seqFISH [17, 18, 19, 20]. This seqFISH approach can detect thousands of mRNA transcripts at single‐cell resolution in a sensitive and effective way in intact tissues, facilitating the mapping of sub‐cellular types and the prediction of cell‐cell interactions [18, 19]. However, its full potential has not been verified in mineralized tissues. Integrating with our previously published scRNA‐seq data [8], we produced a high‐confidence map of the anatomic location of various cell types in normal and arthritic TMJ, uncovered arthritis‐specific cellular neighborhoods and cell‐cell interactions relevant to synovial inflammation and pain, and identified the *Igf1*‐*Il33* axis that drives arthritis and pain, which were validated by mouse functional genetics and provided potential therapeutic targets.

## 2 Results

### 2.1 seqFISH Profiling of Mouse Temporomandibular Joint (TMJ)

**FIGURE 1:**
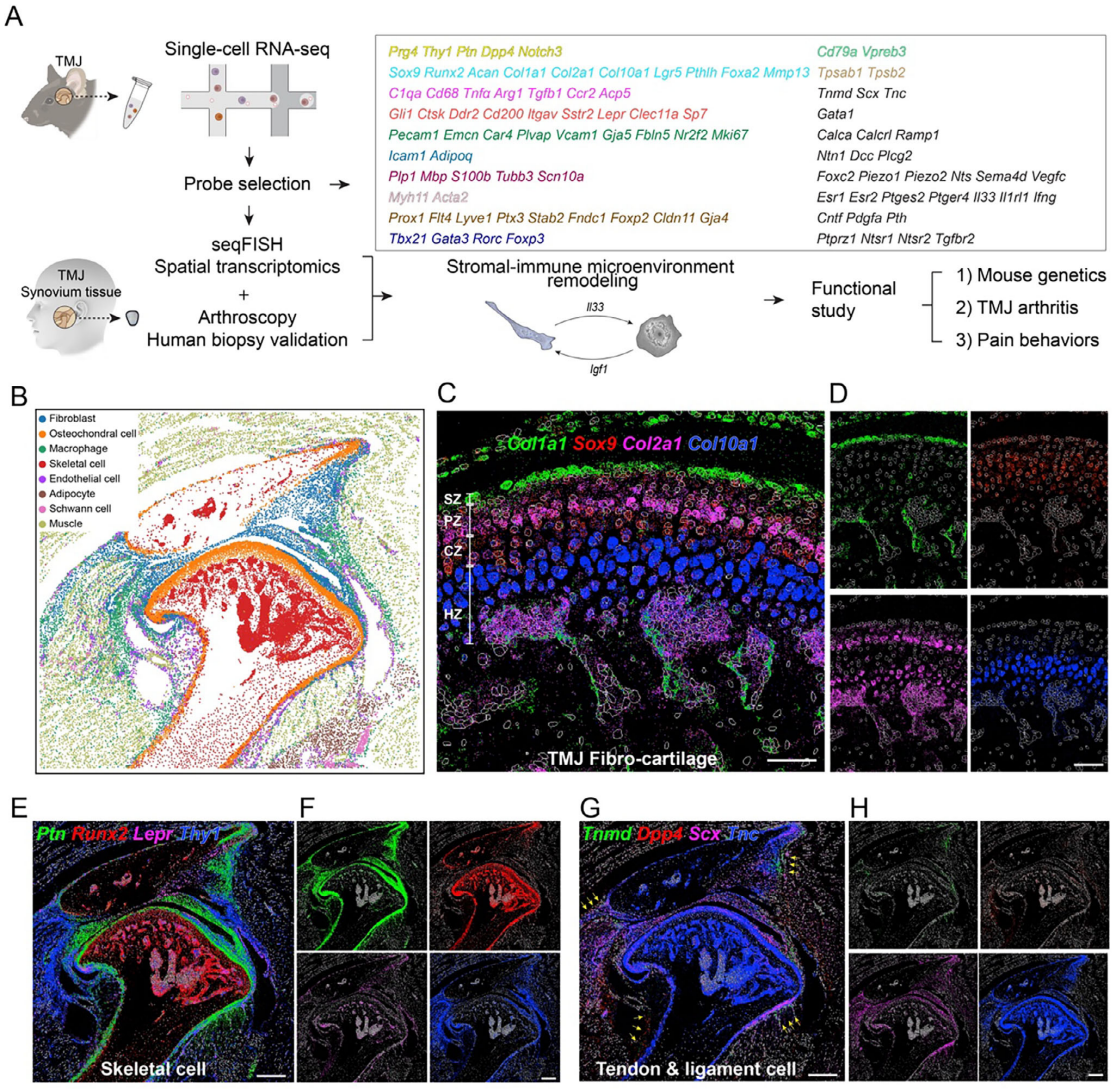
seqFISH profiling of all major cell types and their location in the adult mouse TMJ. (A) Schematics of the overall experimental design. Probes for seqFISH analysis were selected based on marker genes identified in previous scRNA‐seq analysis. (B) Representative seqFISH figures analyzed by Scanpy showing sagittal sections of the 2‐month‐old mouse TMJ under normal conditions. Different colors label different cell types as indicated. (C) Gene expression patterns using SGNlite show the co‐expression of *Col1a1* (green), *Sox9* (red), *Col2a1* (magenta), and *Col10a1* (blue) in the normal TMJ condylar cartilage region, including the superficial zone (SZ), polymorphic zone (PZ), chondroblastic zone (CZ), and hypertrophic zone (HZ). Scale bars: 60 µm. (D) Individual channel of (C). Scale bars: 60 µm. (E) Gene expression patterns produced using SGNlite show *Ptn* (green), *Runx2* (red), *Lepr* (magenta), and *Thy1* (blue) expression in skeletal cells. Scale bars: 300 µm. (F) Individual channel of (E). Scale bars: 300 µm. (G) Gene expression patterns produced using SGNlite show *Tnmd* (green), *Dpp4* (red), *Scx* (magenta), and *Tnc* (blue) expression in tendon and ligament cells. Scale bars: 300 µm. (H) Individual channel of (G). Scale bars: 300 µm.

To delineate the cellular landscape of the adult mouse TMJ, we performed spatial transcriptomics using seqFISH [18, 20], a high‐resolution, probe‐based technique enabling single‐cell gene expression analysis in situ. A total of 96 target genes were selected based on cell‐type or cell‐status markers identified in prior scRNA‐seq studies [8] of control and arthritic TMJ tissues (Figure 1A). Marker genes were chosen to represent major cell types and subpopulations, including fibroblasts (*Prg4*, *Thy1*, *Ptn*), fibrocartilage (*Col1a1*, *Sox9*), chondrocytes (*Acan*, *Col2a1*), hypertrophic chondrocyte (*Col10a1*), macrophages (*C1qa*), monocytes (*Ccr2*), osteoclasts (*Acp5, Ctsk*), skeletal progenitor cells (*Gli1*, *Itgav*, *Lepr*), osteoblasts (*Sp7, Runx2*), vascular endothelial cells (*Pecam1*, *Emcn*, *Vcam1*), adipocytes (*Icam1*, *Adipoq*), Schwann cells (*Plp1*, *Mbp*, *S100b*), muscle cells (*Myh11*, *Acta2*), lymphatic endothelial cells (*Prox1*, *Flt4*, *Lyve1*), T cells (*Tbx21*, *Gata3*, *Rorc*), B cells (*Cd79a*, *Vpreb3*), mast cells (*Tpsab1*, *Tpsb2*), tendon cells (*Tnmd*, *Scx*, *Tnc*), and erythrocytes (*Gata1*). Additional genes of interest were included for exploratory analyses (Table S1 and Figure S1). All seqFISH gene expression data are publicly available via FaceBase to support the construction of a spatial transcriptomic atlas of the mouse TMJ (DOI: 10.25550/8Q‐3KAR).

After generating a curated panel of 96 marker genes for seqFISH‐based spatial transcriptomics, we first analyzed the mouse normal TMJ. By assessing basic metrics using Scanpy [21], the normal TMJ dataset consisted of 62 105 single cells, with an average of 134 ± 185 (mean ± SD) total transcripts and 20 ± 13 individual genes detected per cell. After determining filtering cutoffs using a statistical method commonly applied in scRNA‐seq quality control (median ± ratio ^*^ MAD) [22], we applied minimum and maximum thresholds of 20 and 3000 total transcripts per cell, respectively, and excluded cells with fewer than 10 detected genes. Following stringent quality control, we retained 45 036 cells for subsequent analysis. We performed differential gene expression analysis across clusters to assign cell‐type identities based on established marker gene expression. Consequently, this analysis revealed all major TMJ cell populations, including fibroblasts (*Prg4*⁺/*Thy1*⁺/*Notch3*⁺), osteochondral cells (*Sox9*⁺/*Acan*⁺), macrophages (*Cd68*⁺/*C1qa*⁺), skeletal cells (*Sp7*⁺/*Runx2*⁺/*Cd200*⁺), endothelial cells (*Emcn*⁺/*Pecam1*⁺/*Plvap*⁺), adipocytes (*Adipoq*⁺), Schwann cells (*Mbp*⁺/*Plp1*⁺/*S100b*⁺), and muscle cells (*Myh11*⁺/*Acta2*⁺) (Figure 1B; Figure S2A,B). Because lymphatic endothelial cells (LECs) represent a rare population in a section that was not robustly identified by unsupervised clustering algorithms, we manually annotated LECs based on the co‐expression of *Prox1*, *Flt4*, and *Lyve1*. To analyze the osteochondral cells in greater detail, we extracted and re‐clustered these cells and identified three distinct subpopulations corresponding to perichondrium, chondrocytes, and periosteum based on marker gene expression and spatial localization (Figure S2F–H).

The seqFISH not only enables the broad spatial mapping of major cell types in the TMJ but also provides high‐resolution localization of specific rare subpopulations within the tissue architecture. Consistent with previous findings [23], we observed distinct anatomical zones of condyle cartilage, including a fibroblast‐localized superficial zone (SZ) (*Col1a1*⁺/*Col2a1* ^−^), a polymorphic zone (PZ) with progenitor‐like chondrocytes (*Sox9*⁺/*Col2a1*⁺), a chondroblastic zone (CZ) with flattened chondrocytes (*Col2a1*⁺), and a hypertrophic zone (HZ) with enlarged chondrocytes (*Col10a1*⁺) (Figure 1C,D). *Thy1* marks a population of slow‐cycling stem cells in mouse epidermis regeneration [24, 25] and overlaps with *Ptn* ^+^ cells in the TMJ fibrocartilage surrounding bone and bone marrow stromal cells labeled by *Runx2* and *Lepr*, respectively (Figure 1E,F). These observations raised the possibility that *Ptn* and *Thy1* might mark fibrocartilage progenitor cells in the TMJ. Similarly, *Dpp4*/*Cd26* labels tendon progenitor cells in long bone [26] and is adjacent to the tendon cells labeled by *Tnmd*, *Scx*, and *Tnc* (Figure 1G,H). It is possible that *Dpp4* marks tendon progenitor cells in the TMJ. Together, these analyses provide a comprehensive cellular map of adult mouse TMJ. This foundational work establishes the utility of spatial transcriptomics for dissecting the cellular and molecular architecture of the TMJ.

### 2.2 Mapping of Diverse Cell Types and their Changes in Arthritic Mouse TMJ

**FIGURE 2:**
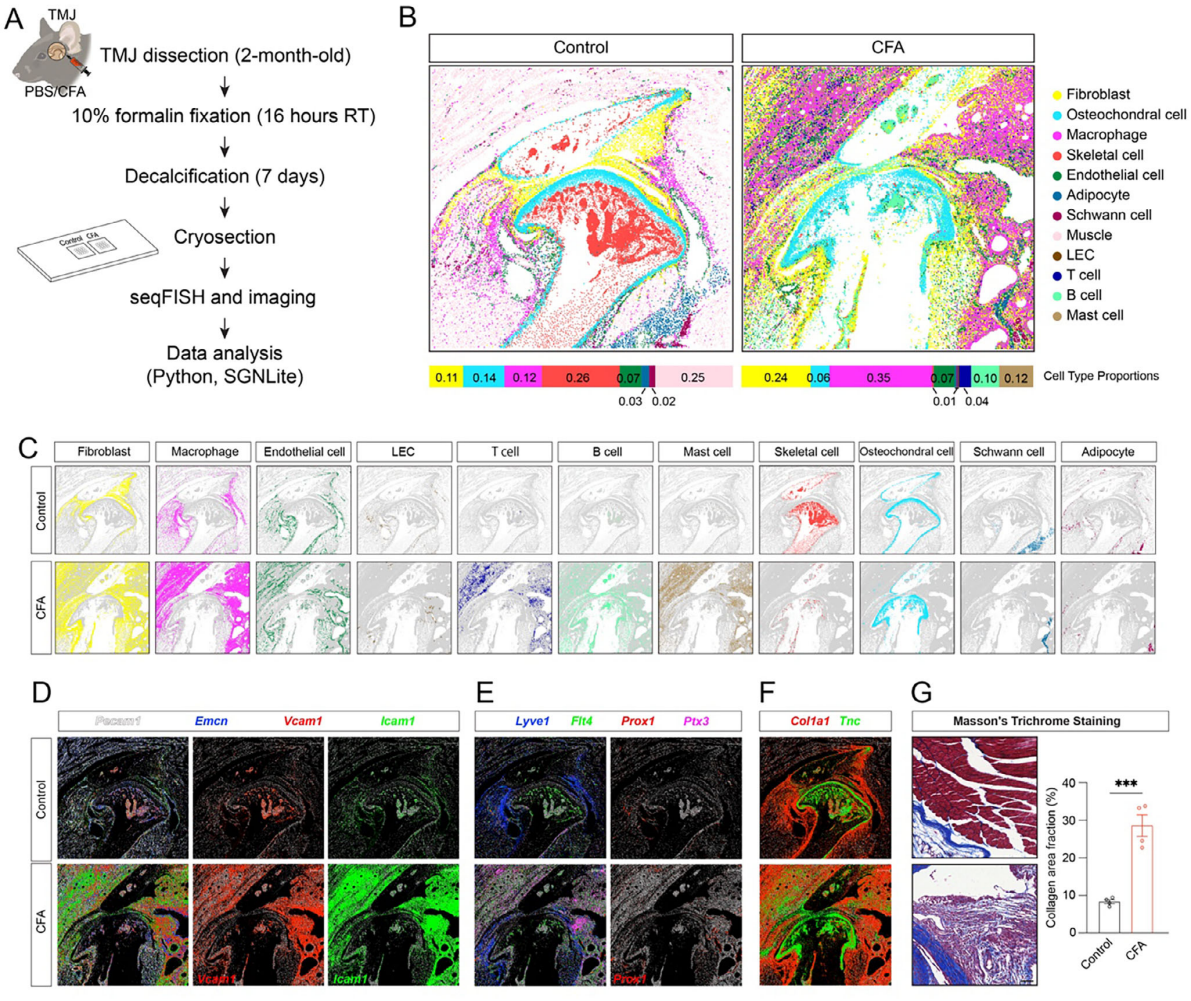
seqFISH mapping of diverse cell types and their changes in arthritic mouse TMJ. (A) Schematic of experimental design. (B) Spatial location of all cell types in control and CFA‐induced arthritic TMJ. Different colors label different cell types as marked. The stacked bar plots below show the cell type proportion. (C) Spatial location of individual cell types between control and CFA‐induced arthritic TMJ. (D) Gene expression patterns using SGNlite show *Pecam1* (white), *Emnc* (blue), *Vcam1* (red), and *Icam1* (green) expression for endothelial cells. Scale bars: 300 µm. (E) Gene expression patterns produced using SGNlite show *Lyve1* (blue), *Flt4* (green), *Prox1* (red), and *Ptx3* (magenta) expression for LECs. Scale bars: 300 µm. (F) Gene expression patterns produced using SGNlite show *Col1a1* (red) and *Tnc* (green) expression for the extracellular matrix (ECM). Scale bars: 300 µm. (G) Brightfield imaging of sagittal sections stained with Masson's Trichrome Staining in the superior regions of control and arthritic TMJ. Scale bar: 100 µm.

To probe the cell number and cell status changes in TMD pathological conditions, we applied seqFISH spatial transcriptomics to the mouse models of inflammatory TMJ arthritis. Complete Freund's Adjuvant (CFA), a well‐established inducer of joint inflammation [27], was intra‐articularly injected into mice to induce TMJ arthritis, recapitulating key features of osteoarthritis pathology and orofacial pain observed in human patients [27]. TMJ tissues were harvested 10 days post‐injection from 6‐week‐old female mice treated with either PBS (control) or CFA (Figure 2A). Based on basic metrics, the arthritic TMJ dataset is comprised of 124 791 single cells, with an average of 139 ± 179 (mean ± SD) total transcripts and 22 ± 14 individual genes detected per cell. After filtering using the same cutoffs as the control, we retained 88 606 cells for downstream analysis. Compared to the control TMJ, the arthritic TMJ exhibited a greater number of cells in similar anatomic regions. Cluster‐based differential gene expression analysis identified all major TMJ cell types observed in the normal condition, along with several additional immune cell populations that emerged under inflammatory arthritis conditions, including T cells, B cells, and mast cells (Figure 2B; Figure S2C,D). Furthermore, we observed an increased abundance of fibroblasts, macrophages, endothelial cells, and LECs, as well as the infiltration of newly recruited immune populations (Figure 2B; Figure S2E). Conversely, osteochondral cells, Schwann cells, and adipocytes were remodeled; skeletal cells were reduced in the arthritic TMJ (Figure 2B,C), consistent with prior histological and molecular characterizations of CFA‐induced TMJ arthritis mouse models [8].

In addition to changes in cell abundance, we observed alterations in cell states and spatial signaling patterns in CFA‐induced mouse arthritic TMJ. First, blood endothelial cells (*Pecam1*⁺/*Emcn*⁺) exhibited elevated expression of *Vcam1* and *Icam1* (Figure 2D), consistent with a reactive venule phenotype that may facilitate leukocyte recruitment [28]. *Icam1*‐ and *Vcam1*‐double‐positive postcapillary venules were described as reactive endothelial venules (REVs) for leukocyte invasion from the blood during inflammation [29]. In the normal TMJ, *Vcam1* expression was primarily localized to subchondral vascular regions, consistent with a marrow‐associated vascular niche. In contrast, CFA‐induced TMJ arthritis showed a marked redistribution of *Vcam1* to synovial vessels, indicating inflammation‐driven endothelial activation at the joint periphery. This spatial shift likely promotes enhanced leukocyte infiltration into the inflamed synovium, contributing to local immune cell accumulation and sustained inflammation. Second, LECs (*Lyve1*⁺/*Flt4*⁺/*Prox1*⁺) within the synovial tissue exhibited increased expression of *Ptx3* (Figure 2E), a marker of inflammatory LEC subpopulation [30]. The *Ptx3* ^+^ LECs are known to facilitate lymphatic expansion by recruiting macrophages with pro‐lymphangiogenic properties [30, 31]. Third, *Col1a1* and *Tnc* expression were both elevated and spatially expanded in the arthritic TMJ (Figure 2F). Their increase in condyle cartilage may reflect aberrant extracellular matrix (ECM) remodeling. Excess deposition of these ECM components in synovial tissue is associated with fibrosis, suggesting a shift toward a fibrotic, ECM‐producing phenotype [32, 33]. These spatial transcriptomic signatures are consistent with Masson's trichrome staining for ECM overproduction (Figure 2G). These results support a model that vascular‐fibroblast interactions promote region‐specific ECM production, remodeling, and fibrosis in arthritic TMJ, potentially exacerbating joint stiffness, orofacial pain, and joint dysfunction.

### 2.3 Synovial Microenvironment Remodeling Coupled with Altered Synovial Cell Fate and Interactions between Macrophages and Fibroblasts in Arthritic TMJ

**FIGURE 3:**
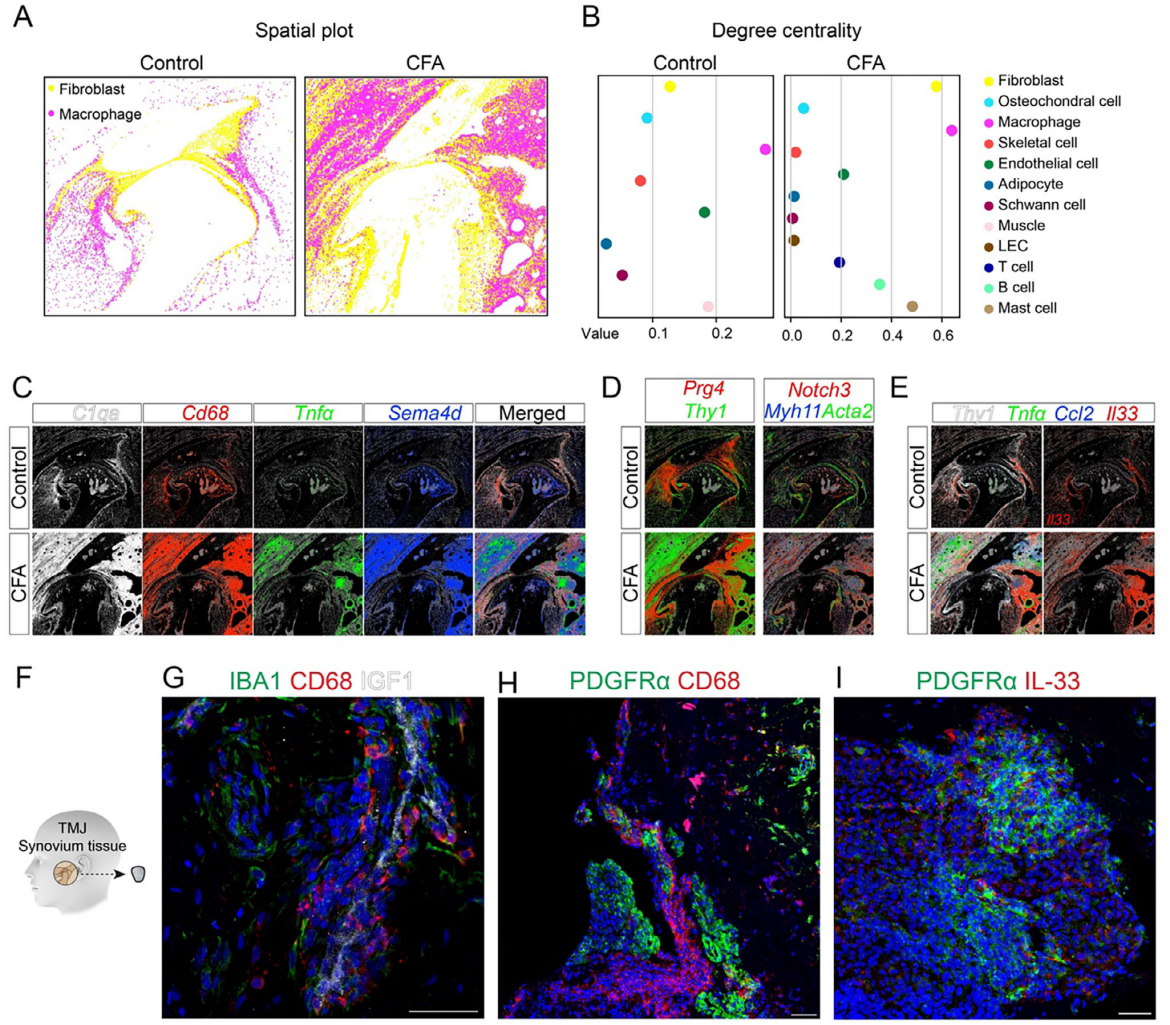
Synovial microenvironment remodeling coupled with altered synovial cell status and interactions between macrophages and fibroblasts in arthritic TMJ. (A) Spatial location of macrophages (magenta) and fibroblasts (yellow) between control and CFA‐induced arthritic TMJ. (B) Dotplot of degree centrality among diverse cell types in control and CFA‐induced arthritic TMJ. Macrophages displayed the highest degree of centrality under both conditions. In the arthritic condition, fibroblasts showed a substantial increase in centrality, second only to macrophages. (C) Gene expression patterns using SGNlite show *C1qa* (white), *Cd68* (red), *Tnfα* (green), and *Sema4d* (blue) expression in macrophages between control and arthritic TMJ. Scale bars: 300 µm. (D) Gene expression patterns using SGNlite show *Prg4* (red) expression in lining fibroblasts; *Thy1* (green) expression in sublining fibroblasts. Scale bars: 300 µm. (E) Gene expression patterns using SGNlite show *Thy1* (white), *Tnfα (green)*, *Ccl2* (blue), and *Il33* (red) expression in fibroblasts between control and arthritic TMJs. Scale bars: 300 µm. (F) Diagram of a human biopsy of TMJ synovium tissue. (G–I) Immunofluorescence staining of human TMJ synovium tissue using antibodies against IBA1 (green), CD68 (red), and IGF1 (white); PDGFRα (green) and CD68 (red) or IL‐33 (red). DAPI stains nuclei (blue). Scale bars: 50 µm.

Inflammation is a hallmark of CFA‐induced TMJ arthritis, and our spatial transcriptomic analysis revealed prominent expansion of both immune and fibroblast cell populations in the arthritic joint. Macrophages were initially restricted to barrier and sublining regions and were drastically expanded throughout the synovial tissue in the arthritic condition (Figure 3A). Synovial fibroblasts similarly showed the most dramatic increase in abundance and spatial coverage in the arthritic TMJ. Beyond qualitative observations, we sought a quantitative understanding of the spatial organization of these cell populations. To this end, we applied Squidpy [34], a spatial analysis toolkit built on Scanpy [21], which provides graph‐based metrics to quantify cellular neighborhoods across tissues. One such metric, degree centrality, treats cell types as nodes and spatial proximities as edges [34, 35]; higher values reflect increased connectivity with surrounding cell types. In the normal TMJ, the three most central cell types were macrophages, muscle cells, and endothelial cells. In contrast, the arthritic TMJ exhibited a shift in cell type proximity, with macrophages, fibroblasts, and mast cells ranking highest in centrality (Figure 3B). Notably, macrophages displayed the highest degree of centrality under both conditions, indicating their consistent spatial proximity to diverse neighboring cell types. In the arthritic state, fibroblasts showed a substantial increase in centrality, second only to macrophages, suggesting enhanced fibroblast‐immune interactions in the inflamed synovium. These findings are consistent with our seqFISH spatial profiling (Figure 3A), highlighting the dynamic remodeling of the TMJ microenvironment during arthritis progression.

In addition to location‐dependent cell number expansion, cell status or cell fate were also changed in the synovial microenvironment in the arthritic TMJ. Macrophages at the control TMJ were not activated, as evidenced by the lack of expression of inflammatory markers *Tnfα* and *Sema4d* as well as macrophage activation marker *Cd68* (Figure 3C). In contrast, seqFISH detected robust upregulation of *Tnfα*, *Sema4d*, and *Cd68* in macrophages of arthritic TMJ synovial tissues, indicating a pro‐inflammatory phenotype. We identified lining fibroblasts marked by *Prg4*, sublining fibroblasts marked by *Thy1*, and vascular fibroblasts labeled by *Myh11*, *Acta2*, and *Notch3*, all of which were expanded in the arthritis synovial tissues (Figure 3D). IL‐33 is a pro‐inflammatory cytokine known to be induced by TNFα [36]. In the fibroblast compartment, arthritic synovial tissues robustly upregulated inflammatory cytokine genes including *Il33*, *Ccl2*, and *Tnfα*, supporting an inflammatory state in synovial fibroblasts (Figure 3E). To assess the translational relevance of these findings, we examined synovial biopsies from patients with painful TMJ arthritis (Figure 3F). Immunohistochemistry confirmed the macrophages in an inflammatory and active status, as evidenced by the CD68 expression in IBA1‐labeled macrophages (Figure 3G). Furthermore, CD68‐labeled inflammatory macrophages were frequently found in close spatial proximity to PDGFRα‐positive fibroblasts (Figure 3H), which is consistent with our identification of a fibroblast‐immune microenvironment in seqFISH (Figure 3A,B). To evaluate the cell status of macrophages adjacent to synovial fibroblasts, we used IL‐33 as an inflammatory marker and found that PDGFRα^+^ fibroblasts frequently express IL‐33 (Figure 3I), suggesting their inflammatory status and conserved fibroblast‐immune microenvironment across species. Together, these findings highlight the synovial cell status changes and altered fibroblast‐immune microenvironment remodeling induced by TMJ arthritis, which might drive inflammation and pain.

### 2.4 Macrophage *Igf1* Deletion Induced Inflammation and Fibroblast Expansion

**FIGURE 4:**
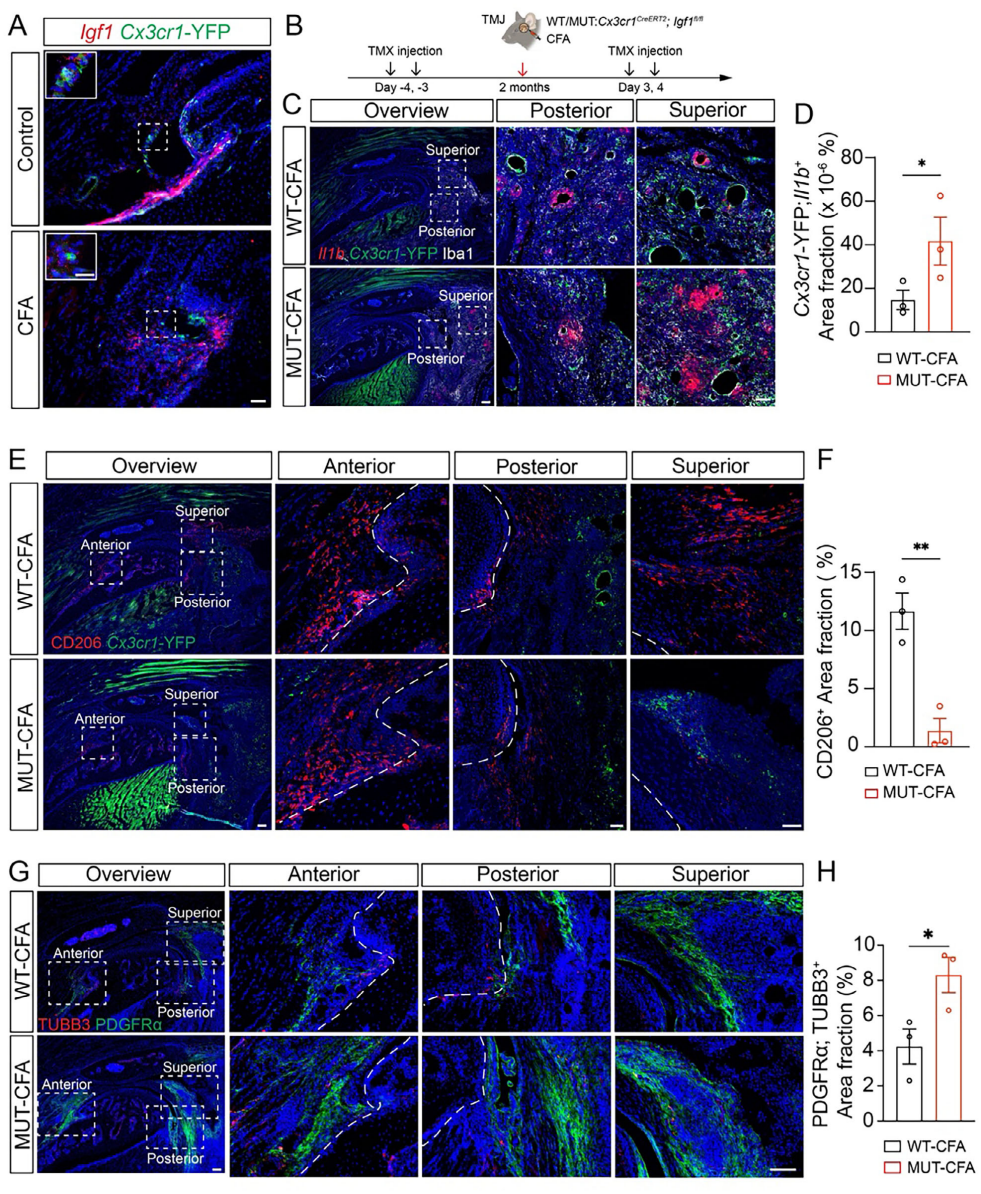
Macrophage *Igf1* deletion leads to inflammation and fibroblast expansion. (A) RNAscope of *Igf1* (red) and immunofluorescence staining of *Cxc3cr1*‐YFP (green) in mouse sagittal anterior TMJ sections. (B) Diagram of experimental design. (C) RNAscope of *Il1b* (red) and immunofluorescence staining of *Cxc3cr1*‐YFP (green) and Iba1 (white) in mouse sagittal TMJ at posterior or superior positions. DAPI stains nuclei (blue). Scale bars: 50 µm. (D) Quantification of the *Cx3cr1*‐YFP^+^; *Il1b* ^+^ area fraction. (E) Immunofluorescence staining of *Cxc3cr1*‐YFP (green) and CD206 (red) in mouse sagittal TMJ. DAPI stains nuclei (blue). Scale bars: 50 µm. (F) Quantification of the CD206^+^ area fraction. (G) Immunofluorescence staining of PDGFRα (green) and TUBB3 (red) in the mouse sagittal TMJ. DAPI stains nuclei (blue). Scale bars: 50 µm. (H) Quantification of the PDGFRα^+^; TUBB3^+^ area fraction. All data are represented as mean ± SEM calculated by Student's *t*‐test, n ≥ 3 mice, ^*^ *p* < 0.05, ^**^ *p* < 0.01.

It has been reported that IGF1 in the macrophage shapes its activation [37]. We hypothesized that macrophages interact with synovial fibroblasts via IGF1 signaling. To test this hypothesis, we first examined *Igf1* expression and found that *Igf1* is expressed by macrophages in mouse TMJ synovial tissues (Figure 4A), which is consistent with human synovial tissue samples (Figure 3G). To investigate the role of macrophage‐derived *Igf1* in modulating fibroblast‐immune interactions during TMJ arthritis, we utilized a macrophage‐specific conditional knockout (cKO) mouse model (*Cx3cr1^CreERT2^*;*Igf1^fl/fl^*) to delete *Igf1* in *Cx3cr1* ^+^ macrophages. To capture the full temporal contribution of Igf1, tamoxifen‐induced deletion was performed continuously across pre‐CFA and post‐CFA (inflammatory) phases (Figure 4B). Efficient *Igf1* deletion in macrophages was confirmed by RNAscope in situ hybridization and immunofluorescence staining (Figure S3A,B).

Iba1 and *Il1b* were used to label macrophages and inflammatory status, respectively. *Cx3cr1^CreERT2^* has built‐in YFP that is often co‐localized with Iba1, further confirming the macrophage identities. Macrophage‐specific *Igf1* deletion led to a significant increase in pro‐inflammatory *Il1b* ^+^ macrophages, as exampled by the analysis at the superior region of the TMJ (Figure 4C,D). Conversely, the number of CD206^+^ anti‐inflammatory macrophages was reduced in mutant TMJ synovial tissues compared to controls (Figure 4E,F), suggesting that *Igf1* is essential for maintaining macrophage phenotypic balance during inflammation. In parallel, we observed an expansion of PDGFRα^+^ fibroblasts in the macrophage‐specific *Igf1* deletion TMJ across anterior, posterior, and superior regions (Figure 4G,H), indicating that loss of macrophage‐derived Igf1 promotes fibroblast activation and proliferation. Importantly, macrophage *Igf1* cKO mice also exhibited enhanced innervation surrounding fibroblast regions within the synovium, as evidenced by increased TUBB3^+^ nerve fiber density (Figure 4G,H). Together, these findings indicate that macrophage Igf1 deletion leads to inflammation and induces synovial fibroblasts and innervation, which might contribute to orofacial pain.

### 2.5 Orofacial Pain Mitigation by Intra‐Articular Injection of IGF1 Protein with Hydrogel in TMJ Arthritis Mice

**FIGURE 5:**
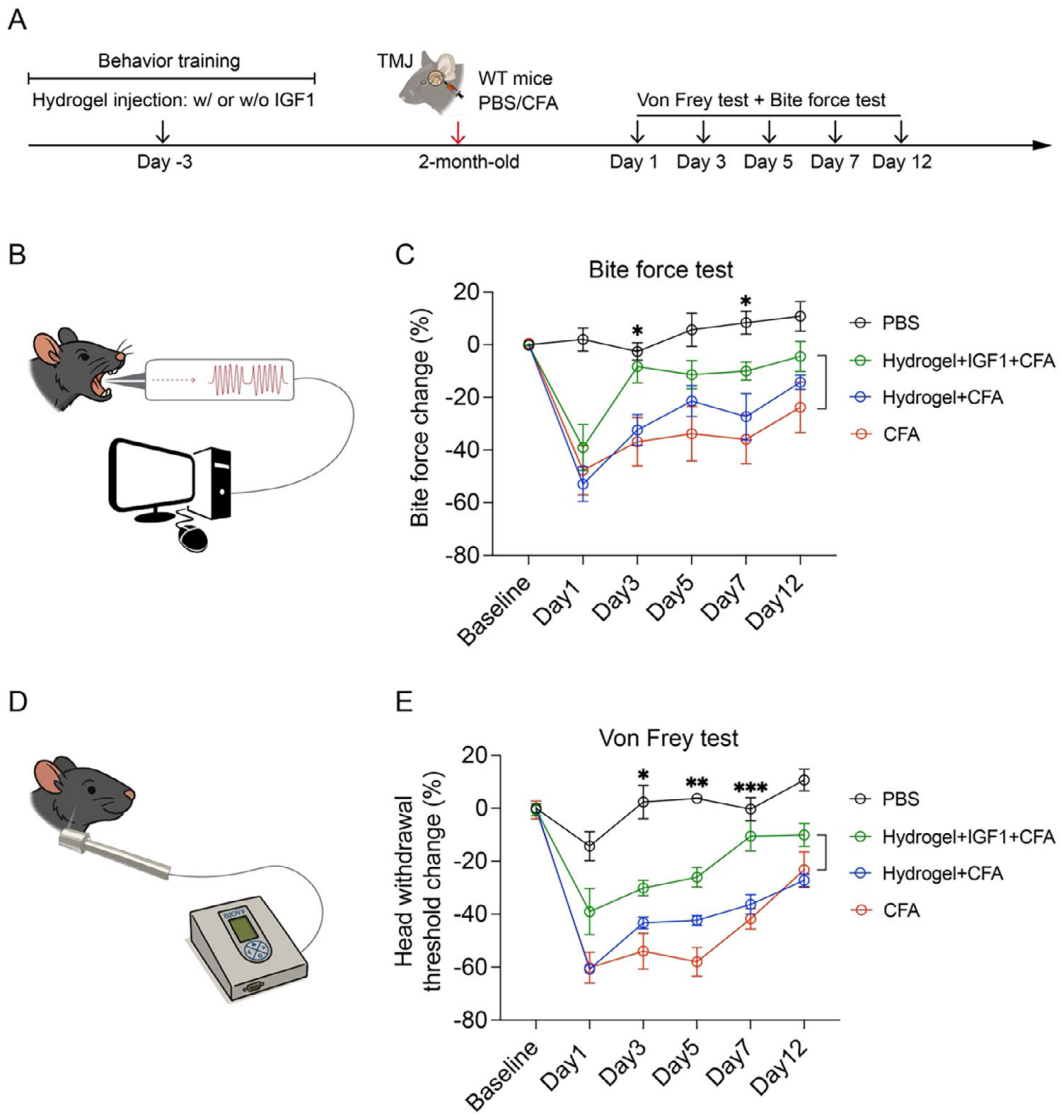
Intra‐articular injection of IGF1 protein with hydrogel mitigates orofacial pain in TMJ arthritis mouse models. (A) Diagram showing that WT mice were injected with vehicle or recombinant IGF1 protein mixed with hydrogel following PBS or CFA injection. Subsequently, pain behavior tests were performed at continuous time points. (B, D) Diagram of bite force and von Frey filament measurement. (C) Quantification of relative bite force values. N = 5 mice (PBS), n = 5 mice (CFA), n = 6 mice (Hydrogel + CFA), n = 10 mice (Hydrogel + IGF1 + CFA). (E) Quantification of head withdrawal threshold. N = 5 mice (PBS), n = 5 mice (CFA), n = 6 mice (Hydrogel + CFA), n = 10 mice (Hydrogel + IGF1 + CFA). All data are represented as mean ± SEM. Two‐way ANOVA with Tukey post hoc test was applied for comparisons among four groups and six time points. ^*^ *p* < 0.05, ^**^ *p* < 0.01, ^***^ *p* < 0.001.

The observation that macrophage *Igf1* deletion leads to increased inflammation and TMJ innervation prompted us to investigate the role of *Igf1* in TMJ orofacial pain. To evaluate the functional role of IGF1 in modulating orofacial pain, we employed a biodegradable, injectable, and sustained‐release hydrogel (*DISH*Gel) for intra‐articular delivery. Specifically, Gel A and Gel B are two liquid components that, when combined, form a hydrogel capable of uniformly incorporating IGF1 protein. Approximately 5 min after mixing, Gel A and Gel B solidify into a stable hydrogel, enabling sustained retention within the TMJ and gradual release of IGF1. Based on this formulation, IGF1 protein was thoroughly mixed with the hydrogel and directly intra‐articularly injected into the TMJ joint space. Due to inflammation and swelling caused by CFA, a second intra‐articular injection post‐CFA was not feasible. Therefore, we performed a single injection of IGF1‐loaded *DISH*Gel first, which does not cause inflammation and tissue swelling. At 3 days post‐IGF1 injection, we induced the inflammatory arthritis mouse models with CFA injection, which is technically durable for this mouse TMJ arthritis model (Figure 5A).

We used bite force and von Frey head withdrawal, two orthogonal assays, to evaluate TMJ function and pain after IGF1 gain‐of‐function. Bite force measurement is an assay to evaluate pain‐related behavior secondary to masticatory function using a force transducer [38, 39, 40] (Figure 5B). Consistent with previous studies [8, 9], CFA induced a robust reduction of bite force compared to the control (Figure 4C) and confirmed TMJ dysfunction and pain behavior in our CFA‐induced inflammation arthritis mouse models. Hydrogel by itself can improve the bite force to a certain degree, but not in a significant manner. In contrast, IGF1, together with hydrogel, significantly increased the bite force in CFA groups (Figure 5C), suggesting the beneficial effects of IGF1 in TMJ function and pain mitigation. The von Frey filament test is a behavioral assay used to assess mechanical sensitivity by measuring the head withdrawal threshold [41, 42, 43] (Figure 5D). There was a robust head withdrawal threshold reduction in the CFA groups with or without hydrogel compared to the control (Figure 5E). IGF1 with hydrogel significantly enhanced the head withdrawal threshold compared to CFA groups (Figure 5E). Together, our longitudinal behavioral assessments suggest that localized delivery of IGF1 protein effectively improves TMJ function and pain‐related behaviors in a mouse model of TMJ arthritis, representing a potential therapeutic strategy.

### 2.6 Inflammatory Macrophage Induced *Il33* Upregulation in Fibroblasts with Inflammation

**FIGURE 6:**
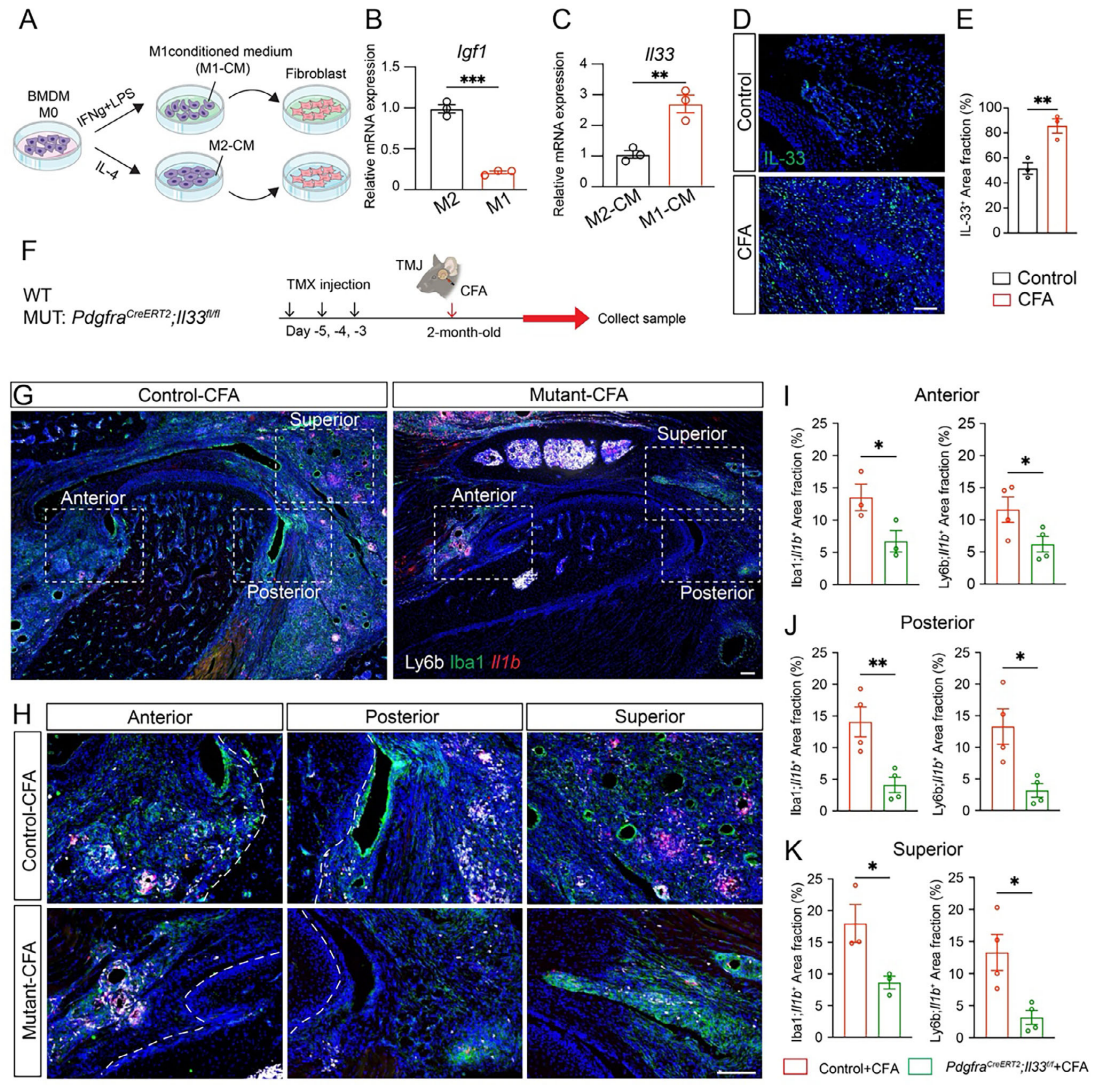
Inflammatory macrophages upregulate *Il33* in fibroblasts that modulate inflammation. (A) Diagrams showing that M1 or M2 macrophage conditioned medium (M1/2‐CM) co‐culture with fibroblasts. (B) qPCR analysis of *Igf1* expression in macrophages, showing significantly lower levels in M1 compared to M2. (C) qPCR analysis of *Il33* expression in fibroblasts showing its higher levels in the M1‐CM treated fibroblasts compared to that exposed to M2‐CM. (D) Immunofluorescence staining of IL‐33 (green) in mouse superior TMJ sections. DAPI stains nuclei (blue). Scale bar: 100 µm. (E) Quantification of the IL‐33^+^ area fraction. (F) Diagram of experimental design on WT and *Pdgfra^CreERT2^; Il33^fl/fl^* cKO mice. (G, H) RNAscope of *Il1b* (red) and immunofluorescence staining of Ly6b (white) and Iba1 (green) in different regions surrounding the TMJ. DAPI stains nuclei (blue). Images in H (20× objective) are enlargements of boxed TMJ areas in G (4× objective) at the anterior, posterior, and superior regions. Scale bars: 100 µm. (I–K) Quantification of the area fraction of *Il1b* ^+^;*Ly6b* ^+^ and *Il1b* ^+^;*Iba1* ^+^ in the anterior, posterior, and superior regions of the TMJ. All data are represented as mean ± SEM calculated by Student's *t*‐test, n = 3 or 4, ^*^ *p* < 0.05, ^**^ *p* < 0.01, ^***^ *p* < 0.001.

To understand why macrophage‐specific deletion of *Igf1* leads to expanded synovial fibroblasts, we hypothesized that macrophage activation reduces *Igf1* levels and contributes to the inflammatory fibroblasts via *Il33* upregulation. To test this hypothesis, we first measured *Igf1* level in inflammatory M1 and anti‐inflammatory M2 macrophages. Quantitative RT‐PCR showed that M1 macrophages expressed significantly lower levels of *Igf1* (Figure 6B), suggesting an *Igf1* reduction in macrophage activation and inflammation. To examine how different inflammatory statuses of macrophages affect *Il33* expression and the cell status of fibroblasts, we performed macrophage‐fibroblast co‐culture experiments. Inflammatory (M1‐like) and anti‐inflammatory (M2‐like) macrophage‐conditioned media (M1‐CM and M2‐CM, respectively) were generated by polarizing bone marrow‐derived macrophages. Fibroblasts were then treated with M1‐CM or M2‐CM for 24 h, after which RNA was extracted for downstream analysis (Figure 6A). Quantitative RT‐PCR revealed that fibroblasts treated with M1‐CM exhibited significantly increased *Il33* expression compared to those treated with M2‐CM (Figure 6C). These findings suggest that low‐*Igf1*‐expressing macrophage activation promotes a pro‐inflammatory fibroblast phenotype characterized by *Il33* upregulation. To validate these findings in vivo, we performed immunohistochemical staining of TMJ tissues from control and CFA‐induced arthritic mice. Consistent with the in vitro data, IL‐33 expression was markedly upregulated in the arthritic TMJ synovial tissues (Figure 6D,E; Figure S3C–F). These results provide a mechanistic explanation for the inflammatory synovial fibroblasts adjacent to activated macrophages, reiterating fibroblast‐immune microenvironment remodeling in TMJ arthritis.

Our studies above suggest that macrophages promote inflammatory fibroblasts via *Il33* upregulation. In turn, fibroblasts might regulate macrophage functions via *Il33*. To test this hypothesis, we performed functional genetic studies and established a genetic mouse model (*Pdgfra^CreERT2^*;*Il33^fl/fl^*) to specifically delete *Il33* in fibroblasts. Fibroblast‐specific IL‐33 deletion was verified by immunofluorescence (Figure S3G,H). We used Iba1 and Ly6b to label macrophages and neutrophils, respectively. Meanwhile, *Il1b* was used to monitor cell inflammatory status. In mice with fibroblast‐specific deletion of *Il33* exposed to CFA‐induced inflammatory arthritis, pro‐inflammatory cytokine *Il1b* expression was reduced in Iba1^+^ macrophages and Ly6b^+^ neutrophils at the anterior, posterior, and superior regions of the TMJ, indicating a dampened inflammatory status (Figure 6G–K). Together, these results suggest that macrophages regulate synovial fibroblasts via *Igf1*, while fibroblasts in turn impact macrophage activation and inflammation via *Il33*, forming a crosstalk loop between synovial fibroblasts and macrophages.

### 2.7 TMJ Innervation and Pain Mitigation Due to Fibroblast *Il33* Deletion

**FIGURE 7:**
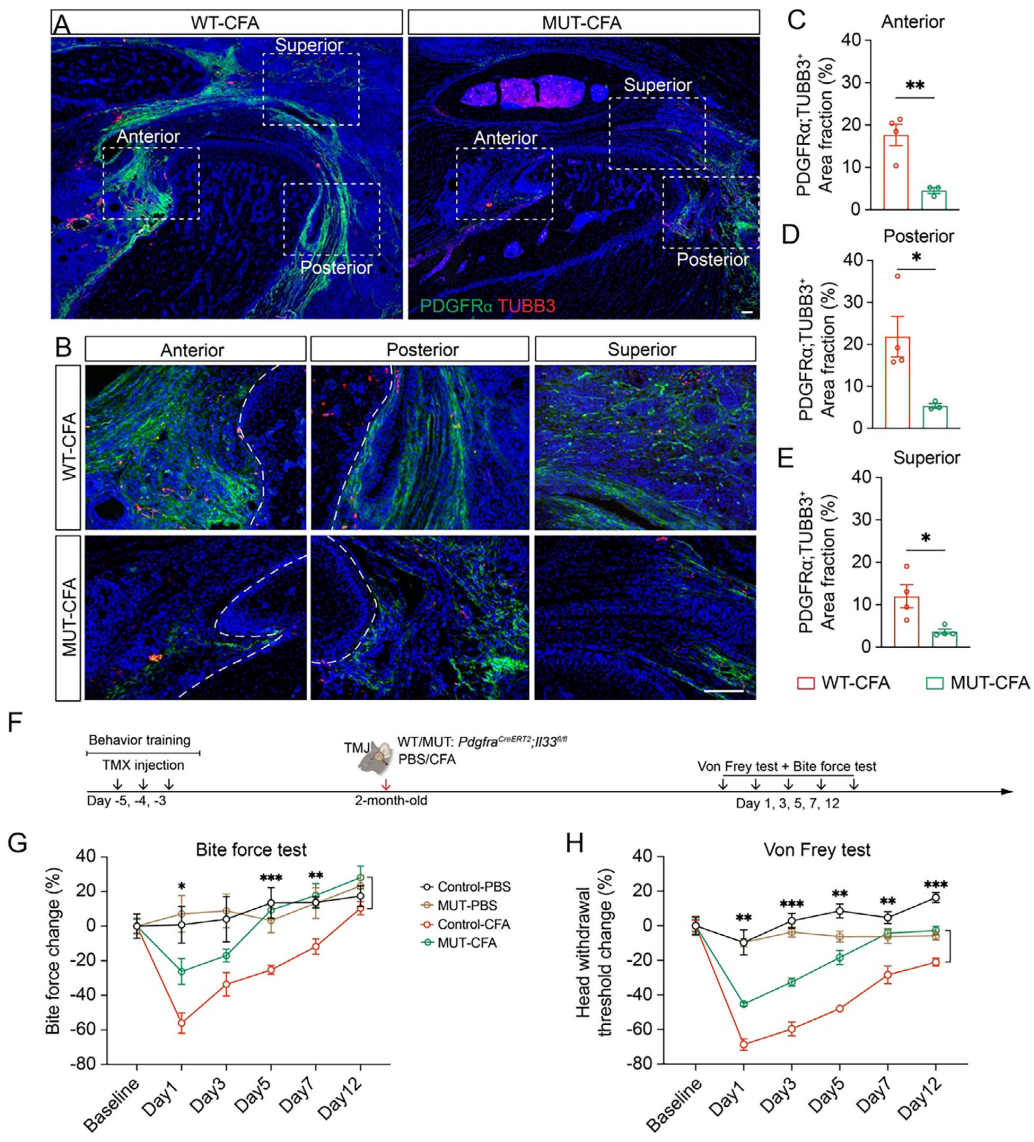
Fibroblast *Il33* deletion mitigates TMJ innervation and pain. (A, B) Confocal imaging of sagittal TMJ sections stained with antibodies against TUBB3 (red) and PDGFRα (green). DAPI stains nuclei (blue). Images in B (20× objective) are enlargements of boxed regions of TMJ in A (4× objective) at the anterior, posterior, and superior positions. Scale bars: 100 µm. (C–E) Quantification of TUBB3^+^; PDGFRα^+^ neuro‐fibroblast area in the TMJ. (F) Diagram of experimental design of pain behavior on WT and *Pdgfra^CreERT2^;Il33^fl/fl^* cKO (MUT) mice. (G) Quantification of relative bite force values in mice. N = 5 mice (Control‐PBS), n = 5 mice (MUT‐PBS), n = 7 mice (Control‐CFA), n = 7 mice (MUT‐CFA). (H) Quantification of head withdrawal threshold in mice. N = 5 mice (Control‐PBS), n = 5 mice (MUT‐PBS), n = 7 mice (Control‐CFA), n = 7 mice (MUT‐CFA). All data are represented as mean ± SEM. Two‐way ANOVA with Tukey post hoc test was applied for comparisons among four groups and six time points, ^*^ *p* < 0.05, ^**^ *p* < 0.01.

There is an increased appreciation of fibroblast‐nerve interaction in pain induction and progression [44]. It is well‐established that neuroimmune regulation is critical for pain generation and regenerative pain medicine [45]. The observation that fibroblast‐specific *Il33* deletion leads to reduced inflammation in TMJ inflammatory arthritis prompted us to examine TMJ innervation and pain in our *Pdgfra^CreERT2^*;*Il33^fl/fl^* arthritis mouse models. Deletion of *Il33* in *Pdgfr* *a* ^+^ fibroblasts led to a marked reduction in fibroblast abundance, as well as TUBB3‐labeled nerve fibers at the anterior, posterior, and superior regions of TMJ arthritis mice (Figure 7A–E), suggesting that fibroblast‐derived *Il33* contributes to aberrant innervation in the arthritic TMJ microenvironment. These findings demonstrated that fibroblast‐derived *Il33* is functionally important to induce innervation in TMJ arthritis.

To evaluate the functional significance of this reduced innervation in *Il33* fibroblast cKO mice, we performed pain behavioral studies using bite force and von Frey tests (Figure 7F). In PBS‐injected control and *Il33* cKO mice, no significant differences in bite force or head withdrawal threshold were observed, indicating that *Il33* deletion does not affect baseline nociception (Figure 7G,H). However, in the CFA‐induced TMJ arthritis model, *Il33* fibroblast cKO mutant mice exhibited significantly higher bite force and head withdrawal thresholds compared to controls exposed to the same CFA treatment, suggesting reduced orofacial pain and improved TMJ function. These results demonstrate that genetically targeting fibroblast‐derived *Il33* effectively mitigates TMJ arthritis‐associated orofacial pain and improves TMJ function, suggesting *Il33* as a promising therapeutic target.

## 3 Discussion

Our spatial transcriptomics on adult mouse TMJ at single‐cell resolution provides anatomical organization of diverse cell types in the jaw joint. We provide a comprehensive view of dynamic changes in the number, status, and neighborhoods of individual cell types in joint arthritis, including LECs, reactive endothelial venules (REVs), fibrotic cells, and various immune cells that were not detected from prior scRNA‐seq studies. Focusing on the most expanded synovial fibroblasts and macrophages, we validated the importance of the microenvironment within synovial tissues in determining gene expression and cell identity. Our functional genetics demonstrated the synovial macrophage and fibroblast crosstalk driving TMJ inflammation and pain via *Igf1*‐*Il33*, which serve as the potential therapeutic targets.

Our studies prove the feasibility of spatial transcriptomics in adult joint at single‐cell resolution. Although spatial transcriptomics studies have been increasingly used in soft tissues [14, 15, 16], it is technically challenging to do so in mineralized tissues due to the difficulty in preserving RNA integrity and tissue morphology following decalcification. To address this, we systematically optimized several key parameters, including the fixation method, decalcification conditions, and tissue age. First, we compared fixation using 10% neutral‐buffered formalin and 4% paraformaldehyde (PFA), both of which yielded comparable results in RNA quality and signal intensity. Further parallel quality control on soft tissues confirmed minimal impact of fixation buffer on the downstream analysis. Second, conventional decalcification protocols use 14% EDTA (pH 7.4) for ≥10 days with solution changes every other day. To improve efficiency, we modified the protocol by using 0.5 m EDTA (∼18%, pH 8.0) for 7 days with daily solution replacement, which preserved RNA quality and enabled successful cryo‐sectioning. Third, we tested different mouse ages and selected 6‐week‐old mice for successful arthritis model development, meanwhile enabling decalcification time reduction and RNA integrity preservation for spatial transcriptomic analysis.

Our studies provide the first comprehensive spatial blueprint of TMJ cell organization, suggesting new potential markers for TMJ stem progenitor cells. Cell‐type identification depends on specific cell‐type markers, whereas individual markers are often not specific enough to exclusively define certain cell types. Therefore, we combine multiple cell‐type markers with anatomic structure to define different cell types in the TMJ [8, 13]. For example, *Sox9* and *Col2a1* are used to define the flattened chondrocytes, *Col10a1* for hypertrophic chondrocyte, and *Col1a1* for fibrocartilage in conjunction with their anatomic locations [23, 46]. Within the fibrocartilage, we found that *Thy1* and *Ptn* localize to the proliferating zone in addition to synovium as well‐established markers for sublining synovial fibroblasts [47]. *Thy1* has been reported as a mesenchymal stem cell (MSC) marker or MSC regulator for tissue regeneration [24, 25]. These observations suggest that *Thy1* or *Ptn* might mark fibrocartilage progenitor cells in the TMJ, which should be verified in future studies. *Dpp4* has been known as the marker for scar‐forming fibroblasts [48]. A *Dpp4*/*Cd26*‐positive tendon progenitor cell population was found in the Achilles tendon for tendon repair [26]. Our seqFISH identified *Dpp4*‐positive cells adjacent to tendon cells labeled by *Tnmd*, *Scx*, and *Tnc* in the TMJ. It is reasonable to speculate that *Dpp4* marks tendon and ligament progenitor cells in the TMJ. The presence of potential progenitor cell markers such as *Thy1*, *Ptn*, and *Dpp4* in TMJ condylar cartilage raises questions about how these progenitor cells are regulated within the local matrix environment. Previous studies have shown that type V collagen not only guides the assembly of collagen I fibrils but also plays a critical role in progenitor cell mechanics within the fibrous layer [49, 50]. Future studies should explore how collagen V interacts with these progenitor cells to support fibrocartilage maintenance and remodeling in the TMJ.

Our studies reveal dynamic changes in diverse cell‐type numbers and cell status induced by TMJ arthritis. In addition to expected bone loss and chondrocyte remodeling, seqFISH uncovers that macrophages, fibroblasts, and blood endothelial cells appear as the most up‐regulated cell types in arthritic TMJ. Meanwhile, arthritis induces a mild increase in LECs, T cells, B cells, and mast cells, which were not captured by prior scRNA‐seq due to their low abundance or drop out during single‐cell dissociation [8]. These immune cells have been reported to increase in arthritic patients [51, 52, 53]. TMJ osteoarthritis (TMJOA) and knee arthritic patients exhibit increased vascular endothelium [54], which occurs in our TMJ arthritis model. Our seqFISH detects a drastically increased population of *Icam1*‐ and *Vcam1*‐double‐positive postcapillary venules, named reactive endothelial venules (REVs). REVs have been reported as the first entry sites of the activated leukocytes from blood in settings of inflammation [29]. We found that *Prox1* is a specific marker for LECs in the TMJ, which is in contrast with its expression in the mesenchymal cells in the meninges [55]. Lymphatic vessels are also labeled by *Lyve1* and *Flt4* and are drastically induced in arthritic TMJ. Future studies should investigate the heterogeneity of the TMJ vasculature and how they contributes to neuro‐immune interaction in arthritis and orofacial pain. Our seqFISH also reveals extensive ECM remodeling toward fibrosis in the inflammatory arthritic TMJ, which is confirmed by Masson's trichrome staining. Knee osteoarthritis (OA) manifests fibrosis in the synovium and infrapatellar fat pad (IFP), which are often correlated with joint pain and stiffness [56]. Adipose tissue is a critical regulator of osteoarthritis fibroblasts that undergo spontaneous adipogenesis, implicating paracrine signaling from fat as a mediator of joint degeneration [57]. Both arthrofibrosis and adipose tissue are barely studied and could play key roles in TMJ arthritis and pain.

Our seqFISH studies suggest that the microenvironment within tissue represents important determinants of both gene expression and cell identities. We improved upon previous scRNA‐seq studies by defining cell clusters and their localization in situ without disrupting the TMJ tissue [8]. We defined the microarchitecture of TMJ synovial tissues by applying computational algorithms in Python (Scanpy and Squidpy) to identify homo‐ and heterotypic cell clusters [21, 34]. In addition to cell number expansion within individual cell types, we found that fibroblasts adjacent to inflammatory macrophages exhibit pro‐inflammatory features, which do not occur in the normal synovial fibroblasts. This observation reflects that the cell environment affects cell fate and is consistent with the notion of fibroblasts as immune regulators [58]. *Prg4*‐ and *Thy1*‐positive cells are lining and sublining synovial fibroblasts, respectively, in knee synovial tissue with distinct functions in joint damage and inflammation [47]. We found that both *Prg4*‐ and *Thy1*‐population are expanded with inflammatory gene profiles in arthritic TMJ synovial tissues. Future genetic labeling can be used to investigate how distinct synovial fibroblast subsets might interact differently with neighboring cells in the microenvironment, driving TMJ arthritis and pain.

Our mechanistic and functional validation of fibroblast‐immune interaction focuses on *Igf1* and *Il33*. *Igf1* and *Il33* expression were detected in activated macrophages and synovial fibroblasts adjacent to macrophages, respectively, which were further confirmed in human TMJ synovial tissues with orofacial pain. In TMJ arthritis mouse models, macrophage‐specific *Igf1* cKO drives macrophages into an inflammatory status and leads to increased synovial fibroblasts with an upregulation of inflammatory factor *Il33*. Conversely, fibroblast‐specific *Il33* cKO mitigates inflammatory macrophages and synovial inflammation. These findings are consistent with reported IGF1 function in shaping macrophage activation and IL‐33 blockage in mitigating knee osteoarthritis [37, 59]. Ectopic IGF1 expression or *Il33* genetic KO improves TMJ function and mitigates orofacial pain in arthritic mouse models, suggesting their functional importance and potential as therapeutic targets. These studies go beyond descriptive characterization and elucidate that spatial transcriptomics‐derived microenvironment mapping has functional significance, pinpointing therapeutic targets for painful TMJ arthritis.

### 3.1 Limitations

While chemically induced TMD models, such as our CFA‐induced TMJ arthritis mice [8, 9], provide valuable insights into inflammatory processes, they have inherent limitations. The acute inflammation triggered by chemical agents cannot fully recapitulate the heterogeneity, biomechanical perturbations, and chronic symptomatology observed in TMD patients. In particular, the CFA model lacks the mechanical stressors central to TMJ pathogenesis. Mechanical models, such as unilateral anterior crossbite (UAC) [60] and bilateral anterior elevation (BAE) [61], can recapitulate relatively well abnormal occlusal loading, subchondral bone remodeling, and altered mandibular kinematics seen in patients. Furthermore, species‐specific differences in immune responses limit the direct translatability of animal findings to human conditions. To address these limitations, future spatial transcriptomic studies can be applied to mechanical, surgical, and genetic models, as well as human patient samples, to better map cellular heterogeneity and microenvironmental interactions underlying TMDs. Our seqFISH relies on the pre‐selected probes from prior scRNA‐seq or bulk RNAseq data. Therefore, it has the drawback of missing the unbiased exploratory discovery based on sequence reads. In this study, our seqFISH failed to detect neuron although specific neuropeptide gene probes such as *Calca* were included, which reflects the general limitation of detecting tube‐like structures due to thin sectioning requirements in spatial transcriptomics. Another caveat is that there are many more ligand‐receptor‐mediated cell‐cell interactions among the diverse cell types in the TMJ. Future studies can include the broad ligand‐receptor genes to gain a comprehensive view of the cellular microenvironment and interactions that mediate arthritis and orofacial pain. Our mouse functional genetics focused on *Igf1* and *Il33* in this study and can be extended to other signaling molecules, which might be potential therapeutic targets for TMJ arthritis and pain.

## 4 Materials and Methods

### 4.1 Mouse Models

All animal experiments were conducted in accordance with the guidelines of the Institutional Animal Care and Use Committee (IACUC), University of Southern California. The C57BL/6J (JAX#000664), *Cx3cr1^CreERT2^* (JAX#021160), *Igf1^fl/fl^* (JAX#012663), *Pdgfra^CreERT2^* (JAX#032770), and *Il33^fl/fl^* (JAX#030619) mouse strains were maintained on a standard rodent diet. All animals were housed under standard conditions and given chow and water ad libitum. Female 6—12‐week‐old mice were used in this study unless otherwise specified. Pain behavioral studies used female mice due to inherent differences between sexes in nociceptive sensitivity, with higher TMJ pain incidence and levels in females.

### 4.2 CFA Intra‐Articular Injection

To locate the TMJ injection site, the zygomatic arch was palpated, and a slight depression approximately 2 mm anterior to the external auditory canal was identified as the anatomical guide. A needle was inserted beneath the posterior portion of the zygomatic arch until it made contact with the underlying bone at an approximate depth of 2 mm. A total of 10 µL of Complete Freund's Adjuvant (CFA; 5 mg/mL, Chondrex, Inc.) was slowly injected into each TMJ capsule bilaterally. After injection, the needle was held in place for at least 5 s before being gently withdrawn to ensure proper delivery. Control animals received bilateral intra‐articular injections of 10 µL sterile phosphate‐buffered saline (PBS) following the same protocol.

### 4.3 Histology Analysis

Mouse TMJ tissues were carefully dissected, fixed, and subjected to decalcification and washing steps. Subsequently, samples were dehydrated through a graded ethanol series, cleared in xylene, and embedded in paraffin for sectioning. Sagittal sections of the TMJ (5 µm thickness) were prepared to assess histological changes. Collagen deposition was evaluated using Masson's Trichrome Staining (Trichrome Stain Kit—Masson's, StatLab, Cat #204183). In the stained sections, cytoplasm, muscle fibers, and keratin appeared in varying shades of pink to red, while collagen fibers were distinctly stained blue.

### 4.4 Immunofluorescence Staining

Mouse TMJ and human synovial tissue sections were used for immunofluorescence staining following standard protocols. Human TMJ synovial tissues were provided by Oral & Maxillofacial surgeon Dr. David Ahn under the guidelines of the University of Southern California Institutional Review Board (IRB). Human synovial biopsies were fixed in 10% formalin at room temperature overnight. Following fixation, tissues were cryoprotected in 30% sucrose at 4°C overnight, then equilibrated in a 1:1 mixture of 60% sucrose and OCT compound at 4°C overnight, and subsequently embedded in OCT on dry ice. For mouse TMJ cryosectioning, joints were first decalcified in 14% EDTA (pH 7.4) for a minimum of 10 days at 4°C. Samples were then cryoprotected by sequential immersion in 30% sucrose overnight, followed by 1:1 60% sucrose/OCT (Tissue‐Tek, Sakura) at 4°C overnight. Tissues were embedded in OCT, frozen on dry ice, and sectioned using a cryostat (Leica CM1850) at 10 µm (human synovial tissue) or 14 µm (mouse TMJ) thickness.

Antigen unmasking solution (Vector, H‐3300) was used for antigen retrieval. The primary antibodies were as follows: Rabbit anti‐Iba1 (FujiFilm, Cat #019‐19741), Rat anti‐CD68 (Bio‐Rad, MCA1957GA), Goat anti‐IGF1 (R&D system, AF‐291), Rabbit anti‐PDGFRα (Cell Signaling Technology, Cat #5241), Goat anti‐IL‐33 (R&D system, AF3625 and AF3626), Rabbit anti‐CD206 (Abcam, ab64693), Rabbit anti‐TUBB3 (Cell Signaling Technology, Cat #5666S), and Rat anti‐Ly6b (Bio‐Rad, MCA771GT). Alexa Fluor 488/568/647 (Invitrogen) were used as secondary antibodies. DAPI (Invitrogen, Cat #62248) was used for nuclear staining. Images were acquired using a Keyence Fluorescence microscope (Keyence, BZ‐X810) or a confocal fluorescence microscope (Leica, STELLARIS). The percentage of positive immunofluorescence signals and area fraction were determined using ImageJ software. Quantification was performed using 3–5 sections per mouse. Each dot in the graph represents the mean value of each sample or mouse within the group. At least three mice from independent mouse litters were analyzed for each group or genotype. Student's *t*‐tests were used for statistical analysis. A significant level was set at a *p*‐value of 0.05.

### 4.5 RNAscope In Situ Hybridization

Sample preparation and RNAscope staining were performed according to standard ACD (Bio‐Techne) protocol with the RNAscope Multiplex Fluorescent v2 (Cat #323100). The probe used for this study was as follows: Mm‐*Il1b* (Cat #316891‐C1). A negative control probe (Cat #320871) was used for staining and imaging to minimize background signals. Quantification of RNAscope staining by ImageJ was performed at 63× original magnification and interpreted according to ACD scoring guidelines.

### 4.6 Nociceptive Behavior Assessment

Baseline measurements of bite force and head withdrawal threshold were obtained during a 3‐day habituation phase preceding intra‐articular injection of PBS or CFA. Following training, most mice initiated biting behavior within 10 s of exposure to the bite force sensor. Animals that failed to exhibit spontaneous biting within 10 s after 3 consecutive days of training were excluded from further testing. For bite force measurement, mice were gently restrained in a modified 50 mL plastic tube that allowed unrestricted and comfortable head movement. Bite force was recorded using a force sensor (YFM‐1‐100, range: 0–100 N) connected to the NBIT RSD‐V2.6.3 software via the NST2000 data acquisition system (Nanjing Shen‐yuan‐sheng Intelligent Technology Co.). Voluntary bite force was recorded over a 2‐min session, and the five highest force amplitudes were averaged to calculate the final bite force value.

Head withdrawal thresholds were assessed using an electronic von Frey analgesiometer. Prior to testing, mice were acclimated in wire mesh cages within the behavior room for at least 1 h. The von Frey filament was applied perpendicularly to the TMJ region, and the withdrawal threshold was defined as the minimal force required to induce a head retraction response. Each mouse underwent a minimum of five independent trials, with 10‐s intervals between each stimulus during approximately 2 min of gentle restraint. The average of all trial measurements was calculated to determine the final head withdrawal threshold. Detailed protocols for the bite force and von Frey assays in the context of TMJ pain have been described in our recent video publication [9].

### 4.7 Tissue Collection and Processing for seqFISH

Mice were sacrificed and perfused with 20 mL cold PBS (Thermo Fisher, AM9625). Tissue surrounding the TMJ structure was collected and fixed in 10% neutral buffered formalin solution (Sigma, HT501128) overnight at room temperature. After that, samples were washed with PBS for 1 h at room temperature and decalcified in 0.5 M EDTA (Thermo Fisher, AM9262) for 7 days at 4°C. Then, samples were dehydrated in 30% sucrose (VWR, Cat #97061‐432) at 4°C overnight, followed by 60% sucrose/OCT (Tissue‐Tek, Sakura) (1:1) at 4°C overnight. Samples were then embedded in OCT compound, frozen on dry ice, and stored at −80°C. Fourteen‐micrometer cryostat sections were cut from embedded TMJ and placed on the functionalized coverslips (Spatial Genomics). Each coverslip has one control and one CFA section within the imageable area. Tissue sections at room temperature were fixed with 4% formaldehyde (Thermo Fisher, Cat #28908) for 15 min, washed with PBS for 5 min three times, dehydrated with 70% ethanol (SIGMA, E7023) for 30 s, air dried within 20 min, and stored at −80°C. A total of three biological samples for each condition were shipped overnight on dry ice to Spatial Genomics Inc. for subsequent RNA quality control and seqFISH experiments [62, 63].

### 4.8 Image Analysis of seqFISH Data

SeqFISH‐based spatial transcriptomic imaging [17] was performed by Spatial Genomics Inc. using a customized gene panel targeting cryosections of PBS‐ and CFA‐treated mouse TMJ samples. Image processing, such as image registration, cell segmentation, and barcode calling, was performed on the Gene Positioning System (GenePS) (Spatial Genomics).

### 4.9 Downstream Computational Analysis of seqFISH Data

#### 4.9.1 Gene Expression Preprocessing

The Python package Scanpy (v1.10.4) [21] was used to process the data. To check basic metrics of the TMJ seqFISH data, AnnData objects [64, 65] were created from the GenePS data. Quality control criteria excluded cells with fewer than 10 expressed genes, fewer than 20 transcript counts, and more than 3000 transcript counts. Cell filtering thresholds were determined using the formula: median ± ratio ^*^ MAD [22], with the ratio parameter adjusted for each sample to normalize variations in total transcript counts and cell size [66]. Then, the data was normalized and scaled using the normalize_total() and log1p() functions.

#### 4.9.2 Clustering and Differential Expression

To identify unsupervised clusters, we first performed Principal Component Analysis (PCA) using all genes as input to extract 50 PCs and generate a neighborhood graph. We then computed a UMAP of the reduced dimensionality. To identify clusters, the Leiden algorithm [67] with a resolution parameter of 0.5–0.8 was performed. For osteochondral cell subclustering analysis, an optimized k‐means approach [68] was used to determine the appropriate number of clusters. Differentially expressed (DE) genes between Leiden clusters were identified using the rank_genes_groups() function, applying the Wilcoxon rank‐sum test. Due to substantial transcriptional shifts induced by CFA treatment, attempts to integrate Control and CFA samples using Harmony or RPCA resulted in overclustering and condition‐specific artifacts, even at low resolution. To preserve biologically meaningful clustering and accurate annotation, downstream analyses were performed separately for each condition.

#### 4.9.3 Cell‐Type Identification

To assign a cell‐type label to each Leiden cluster of seqFISH cells, DE genes for each cluster were compared against a curated list of cell‐type‐specific markers [8] (Figure 1A). Following multiple rounds of clustering, differential expression analysis, and canonical marker evaluation, cell‐type labels were manually annotated by integrating gene expression profiles with anatomical localization within the tissue. Spatial maps of selected cell types were generated based on either these annotated identities or the co‐expression of relevant marker genes. LECs, T cells, B cells, and mast cells could not be identified through unsupervised clustering in the PBS‐treated mouse TMJ cryosection. Therefore, these populations were manually annotated based on the co‐expression of selected marker genes. LECs were defined as cells co‐expressing *Prox1*, *Flt4*, and *Lyve1*. T cells were identified by the co‐expression of *Gata3*, *Tbx21*, and *Thy1*. B cells were annotated as cells co‐expressing *Gata1* and *Cd79a*, while mast cells were defined by the co‐expression of *Tpsab1* and *Tpsb2*.

#### 4.9.4 Spatial Analysis Computing Centrality Scores

The Python package Squidpy [34] was used to quantify cellular neighborhoods within the tissue. To investigate the spatial distribution of macrophages and fibroblasts, the centrality_scores() function was applied. One of the key metrics generated was degree centrality, which models cell types as nodes and their spatial proximities as edges [34, 35]. Higher values indicate greater connectivity with neighboring cell types.

#### 4.9.5 Spatial Mapping of Gene Expression

SeqFISH in situ gene expression patterns were visualized using SGNlite software (v0.0.12; Spatial Genomics) and Scanpy toolkit [21]. Supplementary figures showing individual gene expression in PBS‐ or CFA‐treated mouse TMJ samples were generated side by side, using a shared upper limit for the color scale (vmax = 20) to ensure comparability.

### 4.10 Statistic and Reproducibility

Statistical analyses were performed using GraphPad Prism (version 9.0.0) software. Data were presented as mean values ± SEM. To compare more than two experimental groups, a two‐way ANOVA with Tukey post hoc test was performed, and for comparison between two groups, two‐tailed or one‐tailed (as indicated in figure legends) unpaired Student's *t*‐tests were used to calculate *P* values. Each experiment was repeated independently at least three times with similar results as specified in the figures and figure legends. Graphs showing mean ± SEM for each group represent at least three biological replicates (n ≥ 3 mice, as indicated in figure legends). Images of immunofluorescence staining were representative of at least three biological replicates (n ≥ 3 mice) showing similar results.

## Author Contributions

Z.Y.L., S.J., Y.S., J.Y.C., Q.C., and P.F.K. performed all experiments. Z.Y.L. analyzed all bioinformatic data. F.X.C. prepared a hydrogel. D.A. and Y.Q. prepared human biospecimens. J.F.C. and Z.Z. designed the experiments and supervised the research. J.F.C. and Z.Y.L. co‐wrote the manuscript.

## Funding

This project is supported by grant R01DE033511 (J.C.) from the National Institute of Dental and Craniofacial Research (NIDCR).

## Conflicts of Interest

The authors declare no conflict of interest.

## Supporting information




**Supporting File 1**: advs73489‐sup‐0001‐SuppMat.docx.

**Supporting File 2**: advs73489‐sup‐0002‐TableS1.xlsx.

## Data Availability

The raw and processed single‐cell RNA sequencing (scRNA‐seq) data from PBS‐ or CFA‐treated TMJ have been deposited in the Gene Expression Omnibus (GEO) database under accession number GSE267942. The raw and processed SeqFISH data are available in the FaceBase database under the DOI: 10.25550/8Q‐3KAR.
