# Supplementary material for: Spatial Transcriptomics of TMJ Reveals a Remodeling Fibroblast‐Immune Microenvironment Driving Arthritis Pain
Source: Adv Sci (Weinh). 2026 Jan 7;13(18):e19816. doi: 10.1002/advs.202519816 (PMC13042702; doi:10.1002/advs.202519816)
Supplement: Supplementary file 1 — Supporting File 1: advs73489‐sup‐0001‐SuppMat.docx. [file ADVS-13-e19816-s002.docx]

**Supplementary figures**

**
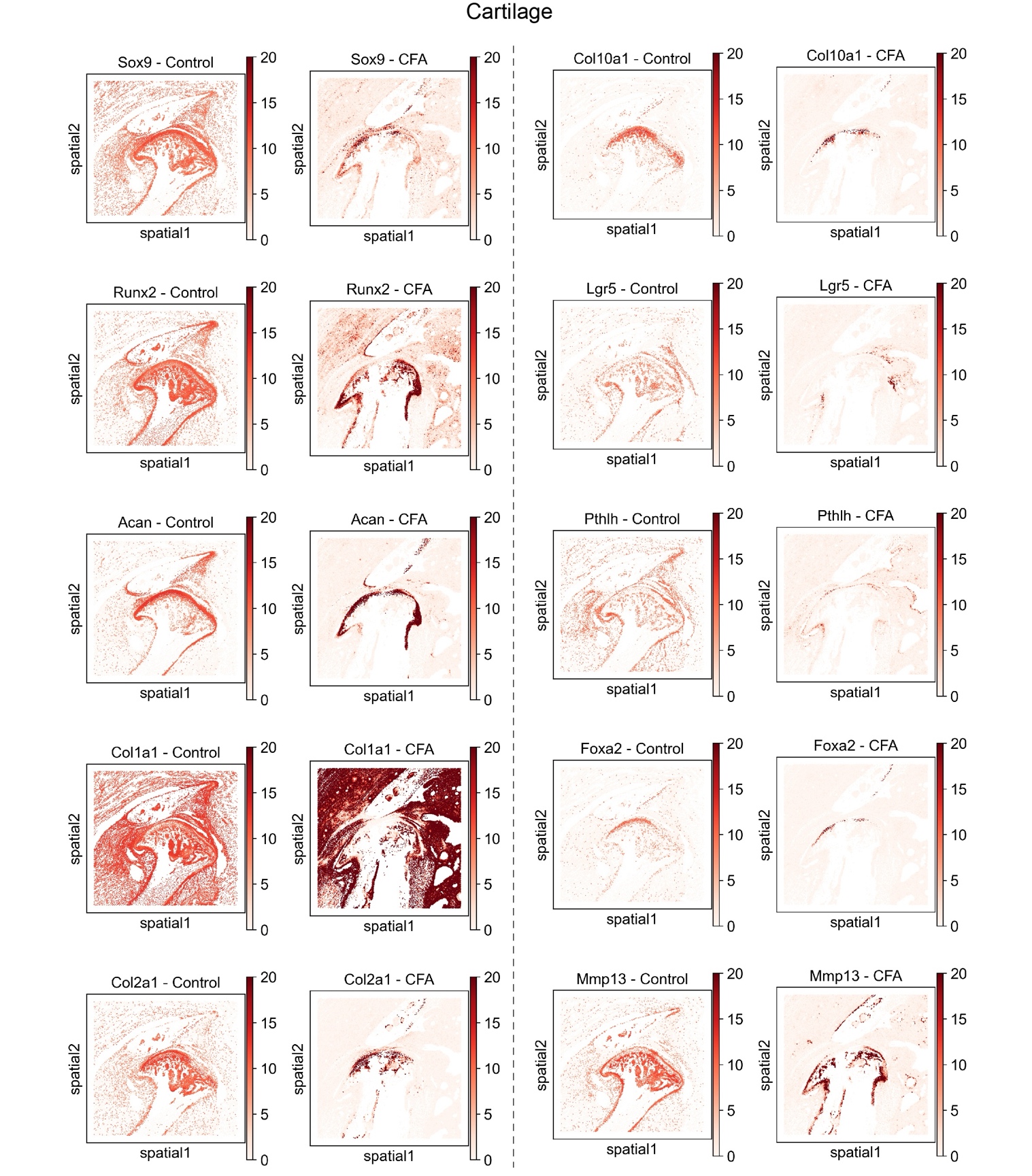
**

**
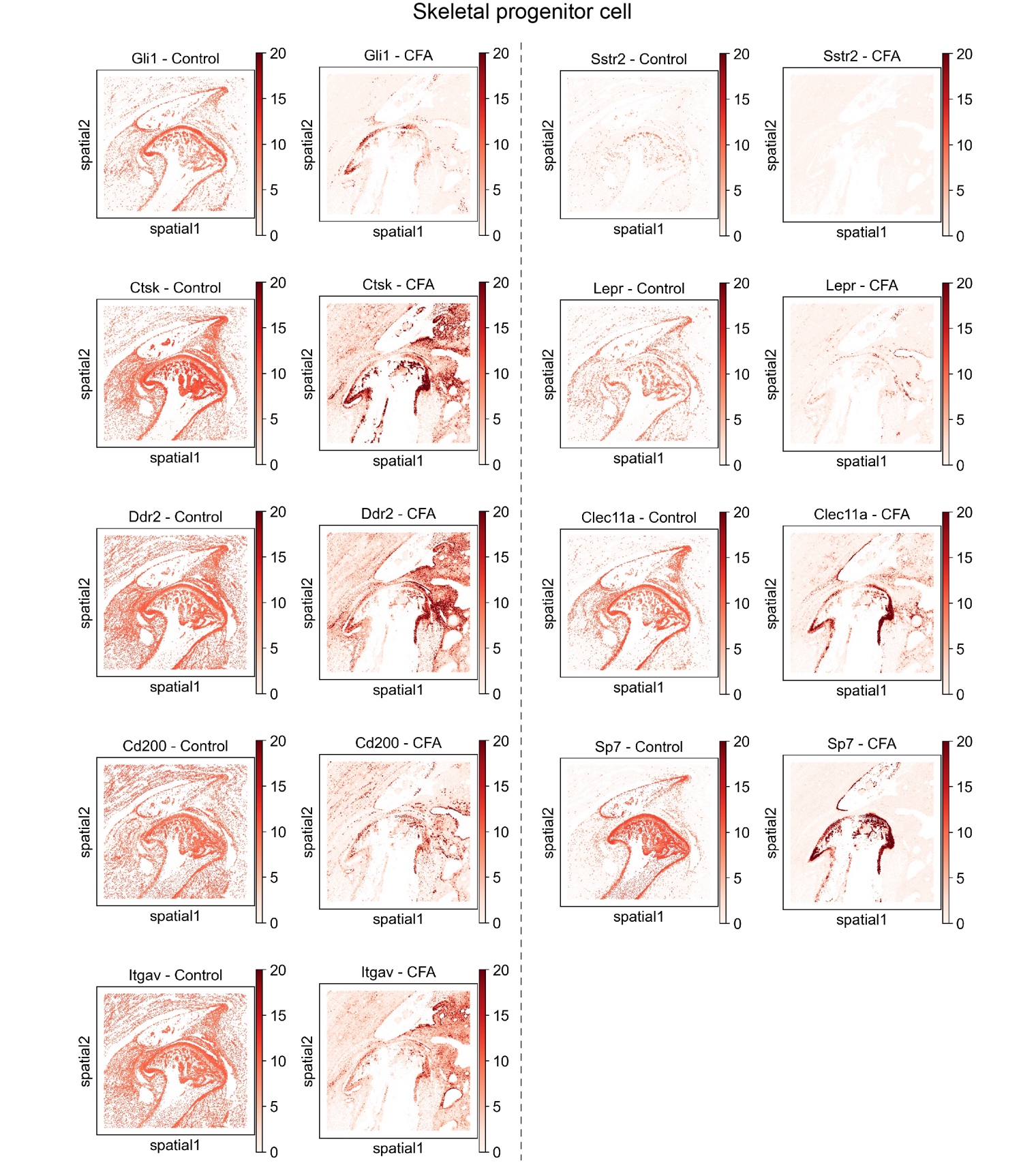

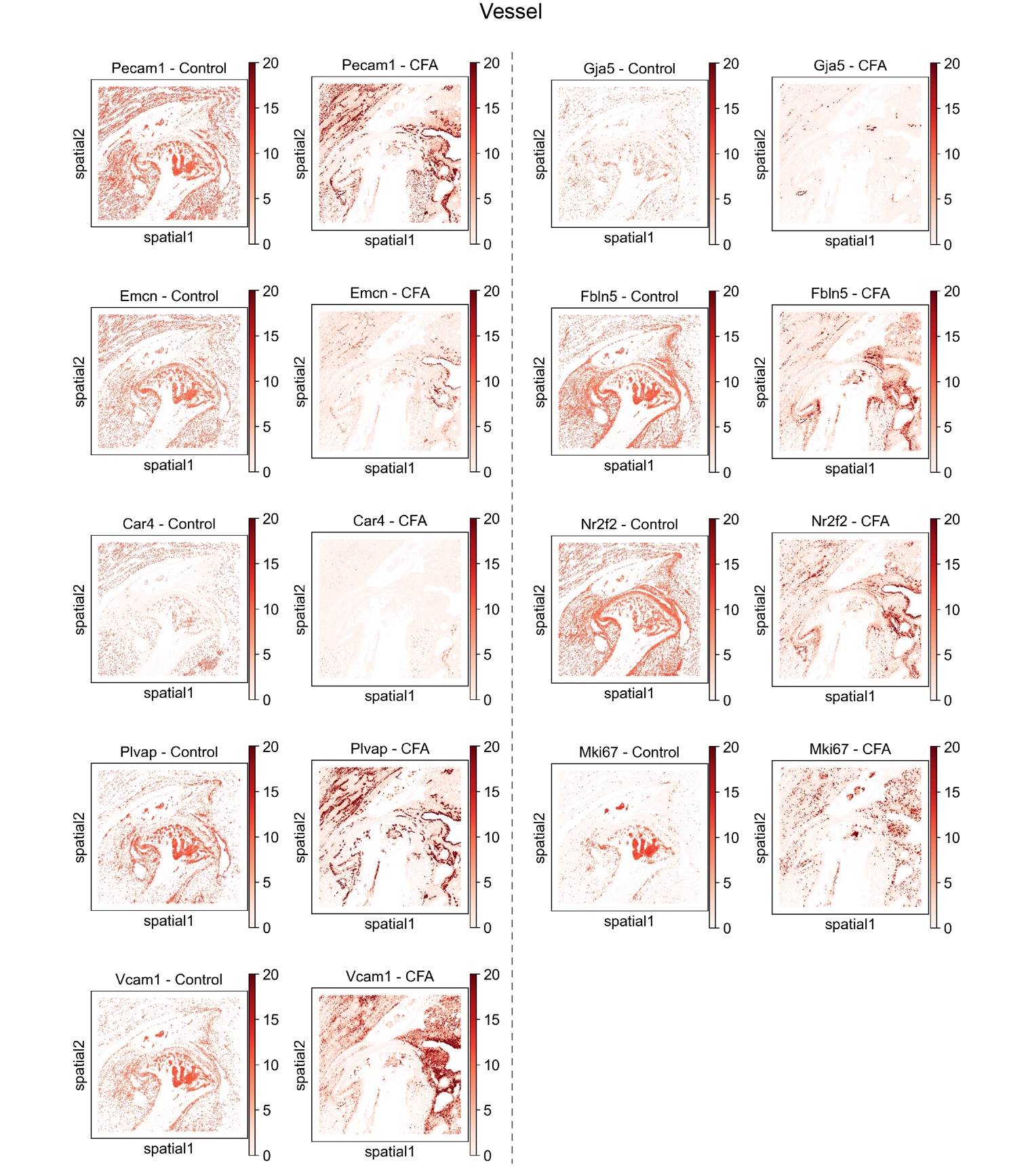
**

**
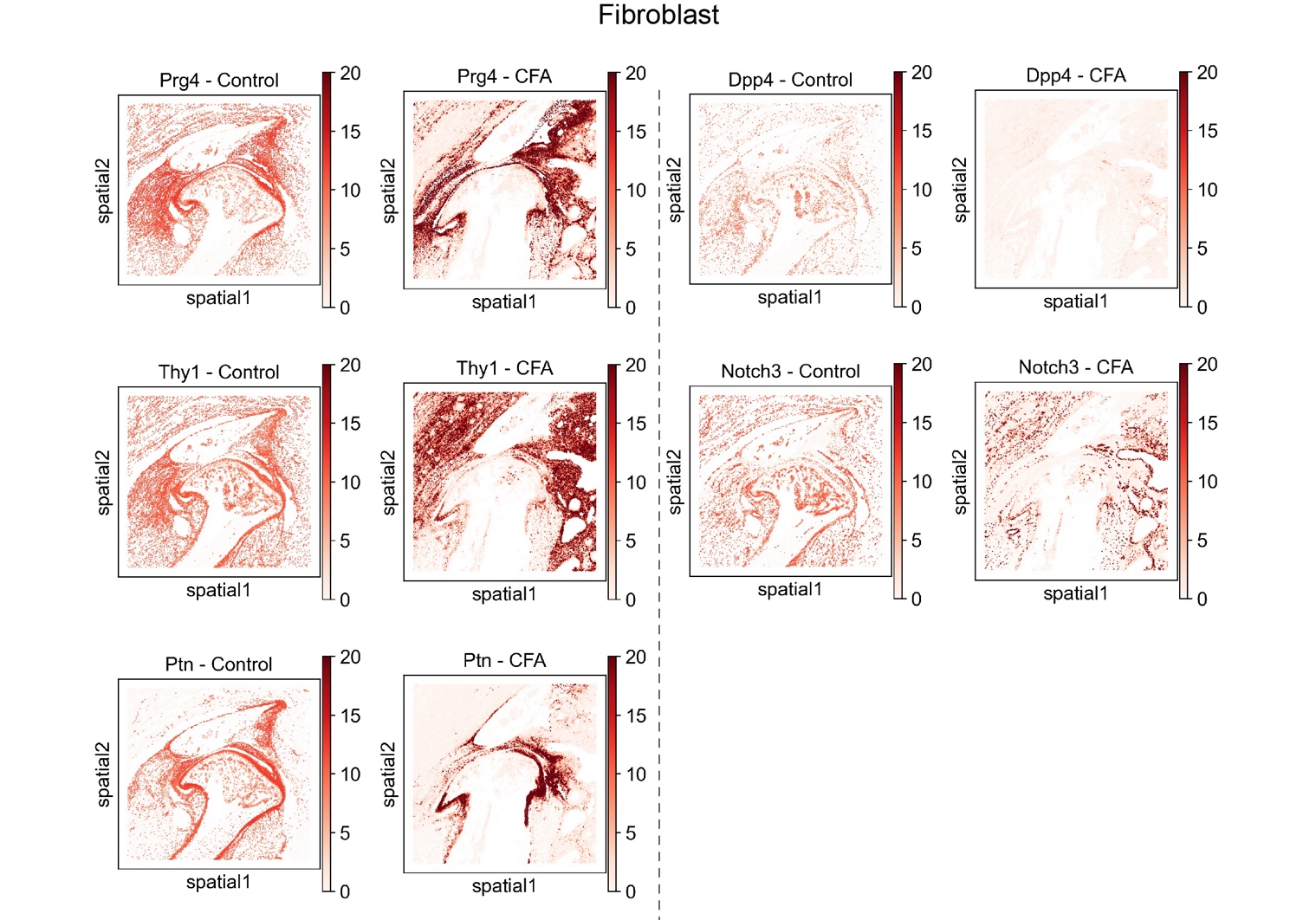

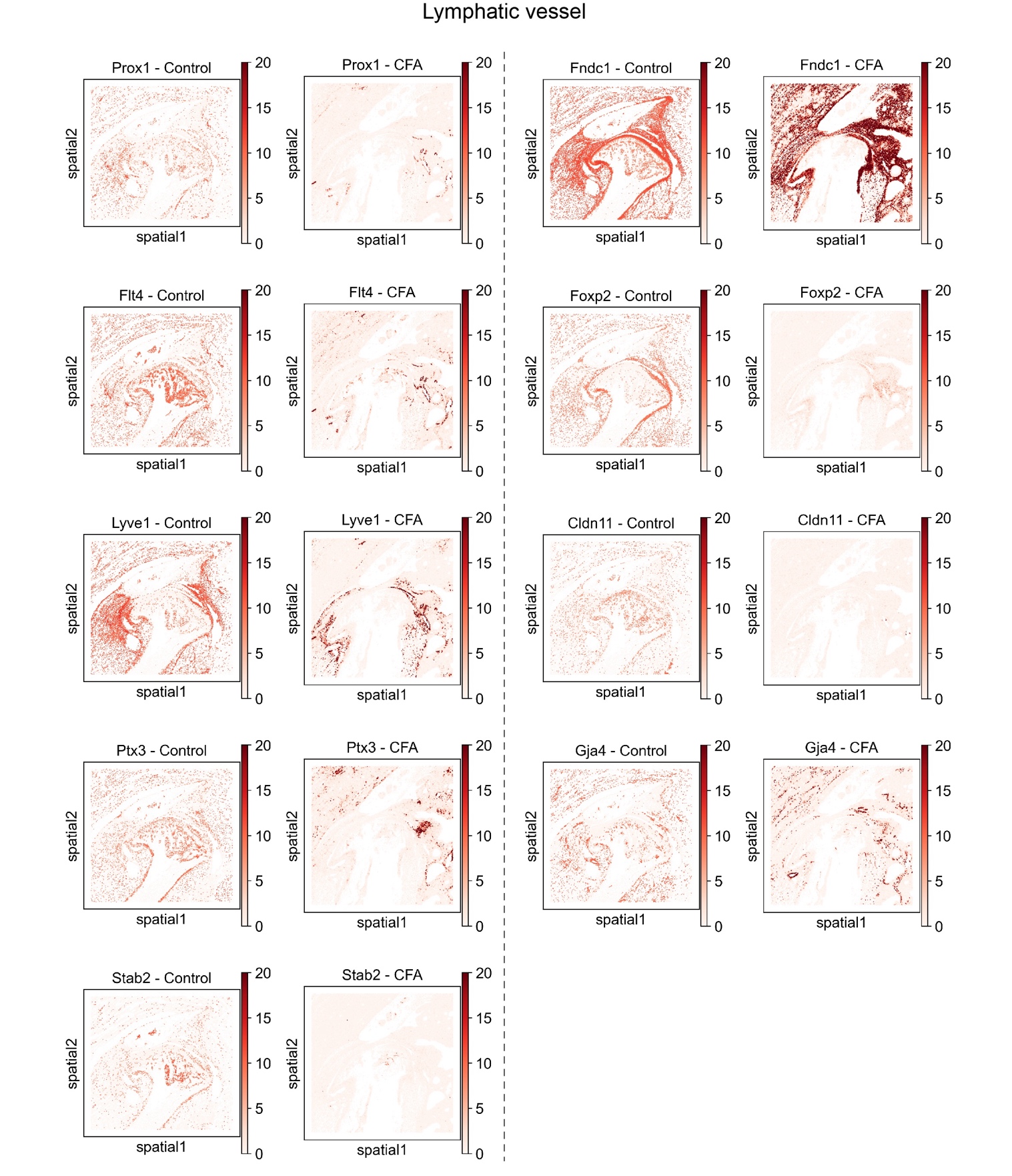

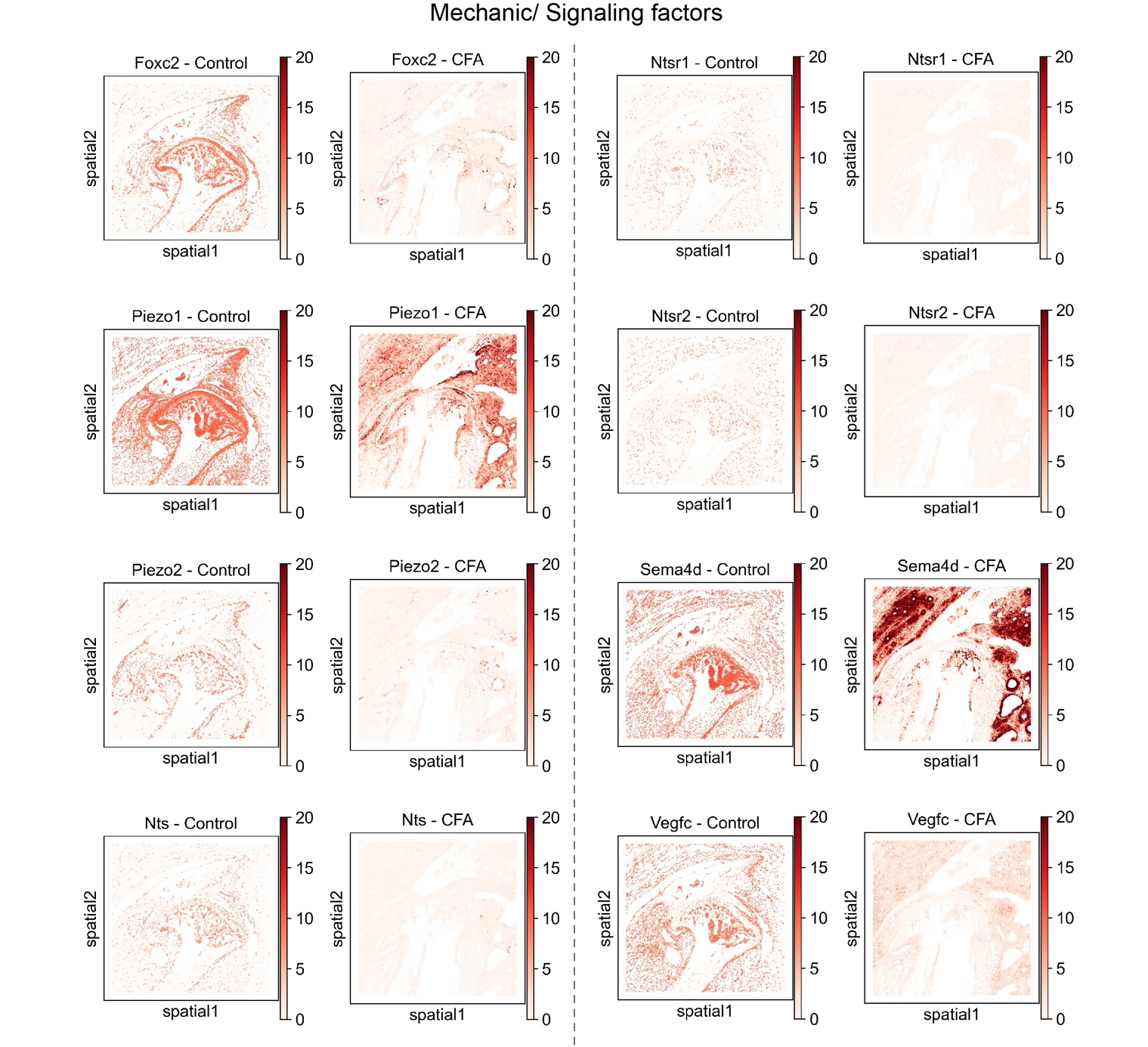

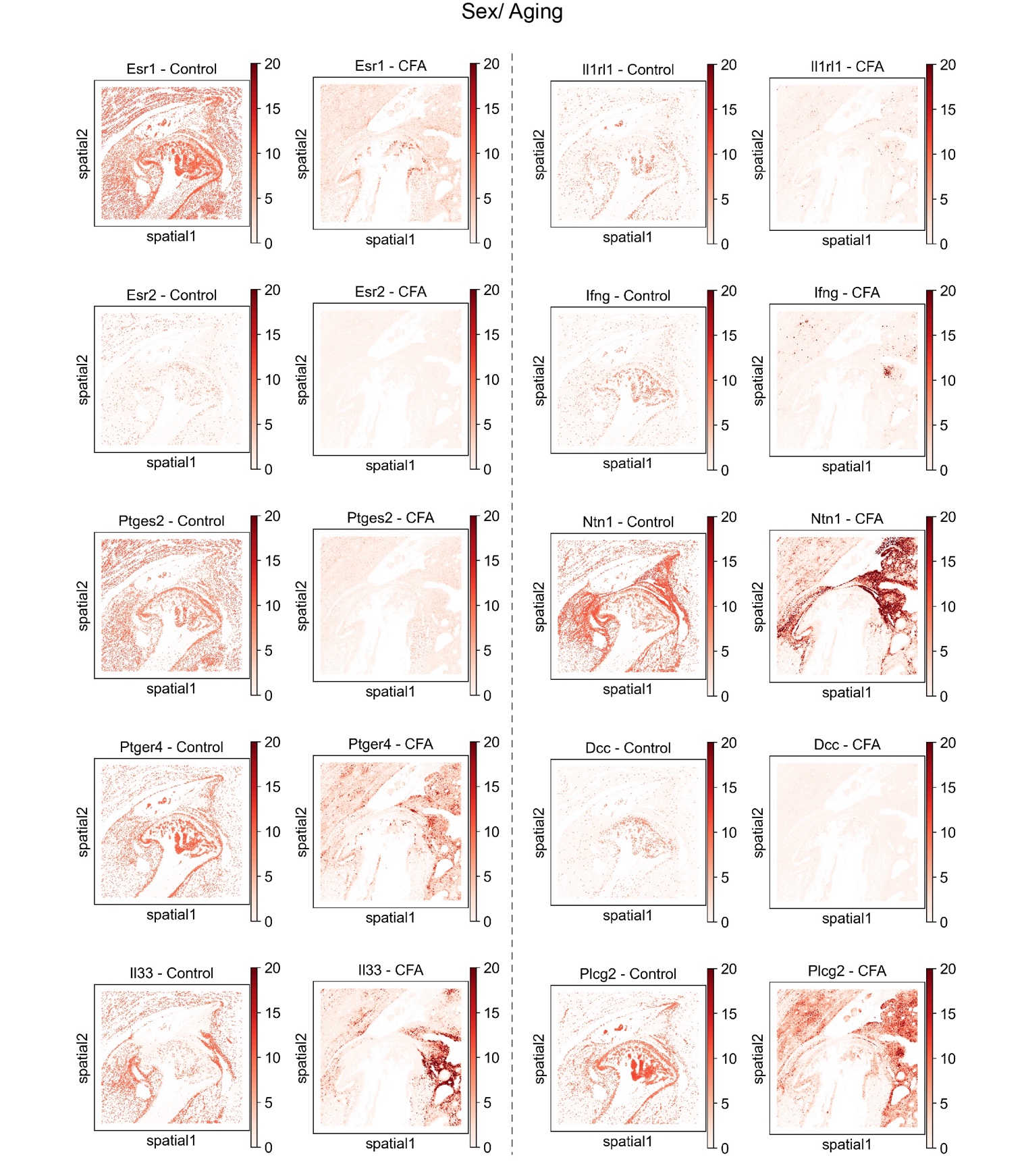

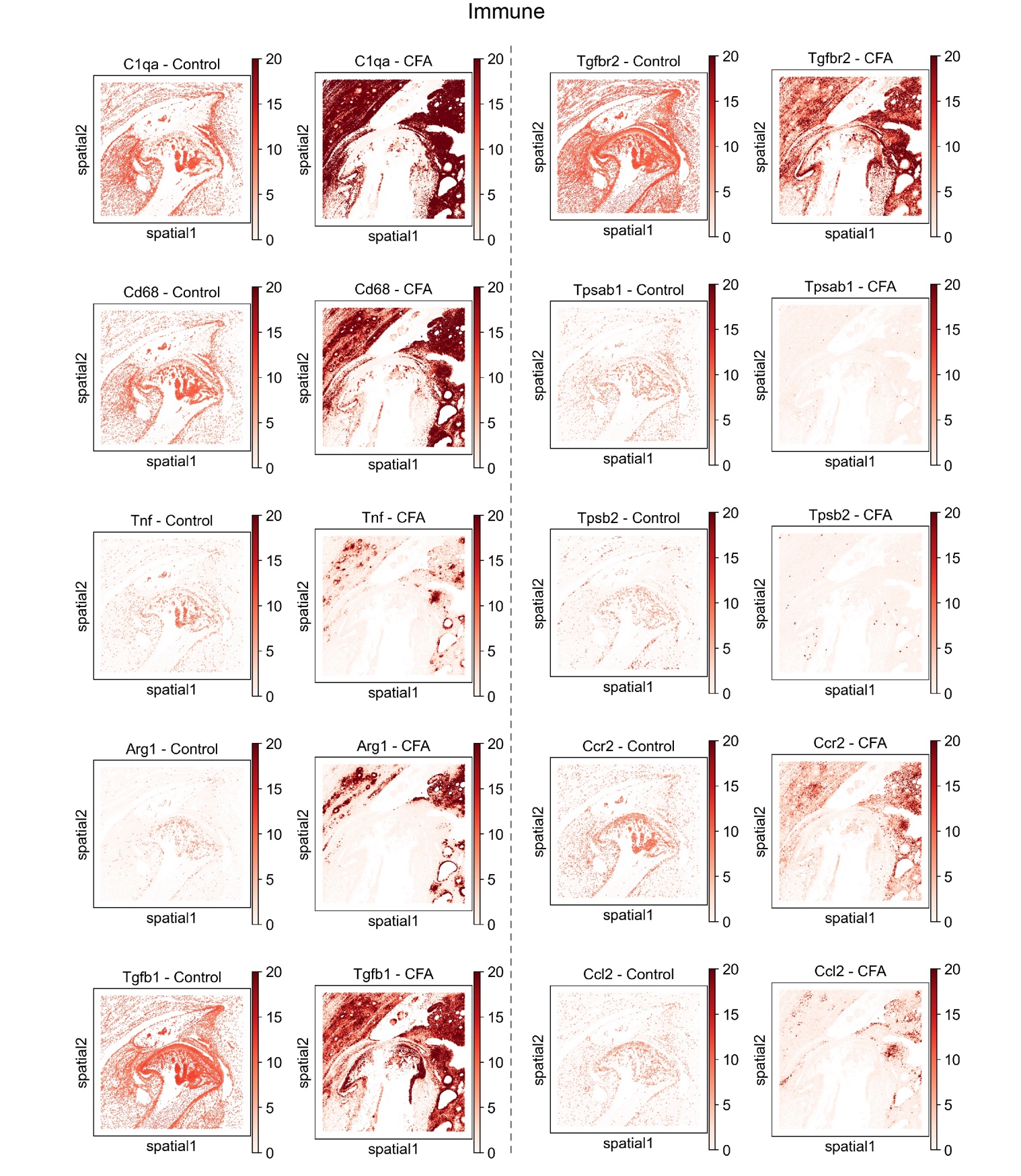

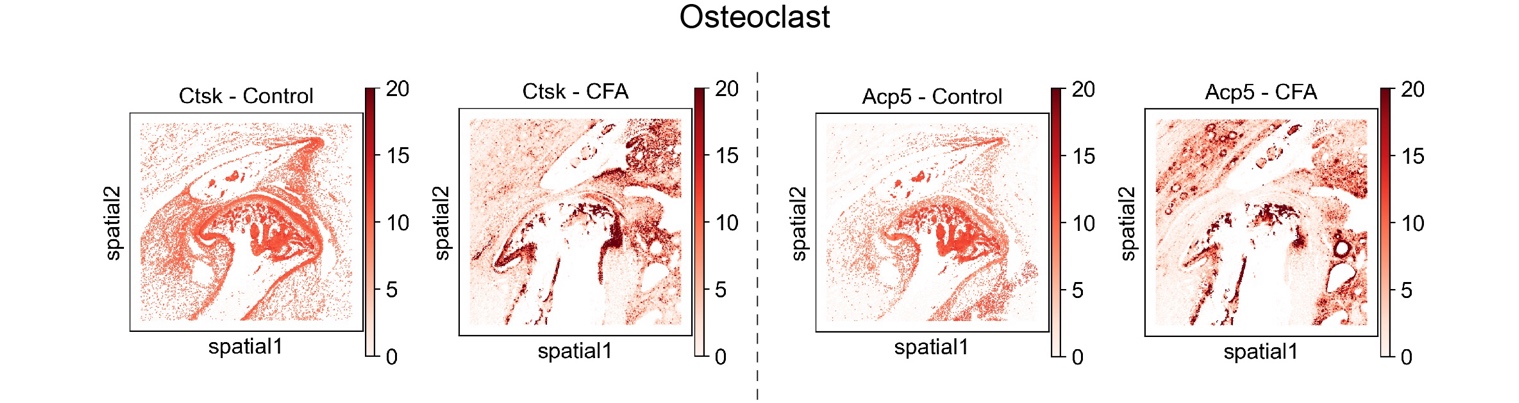

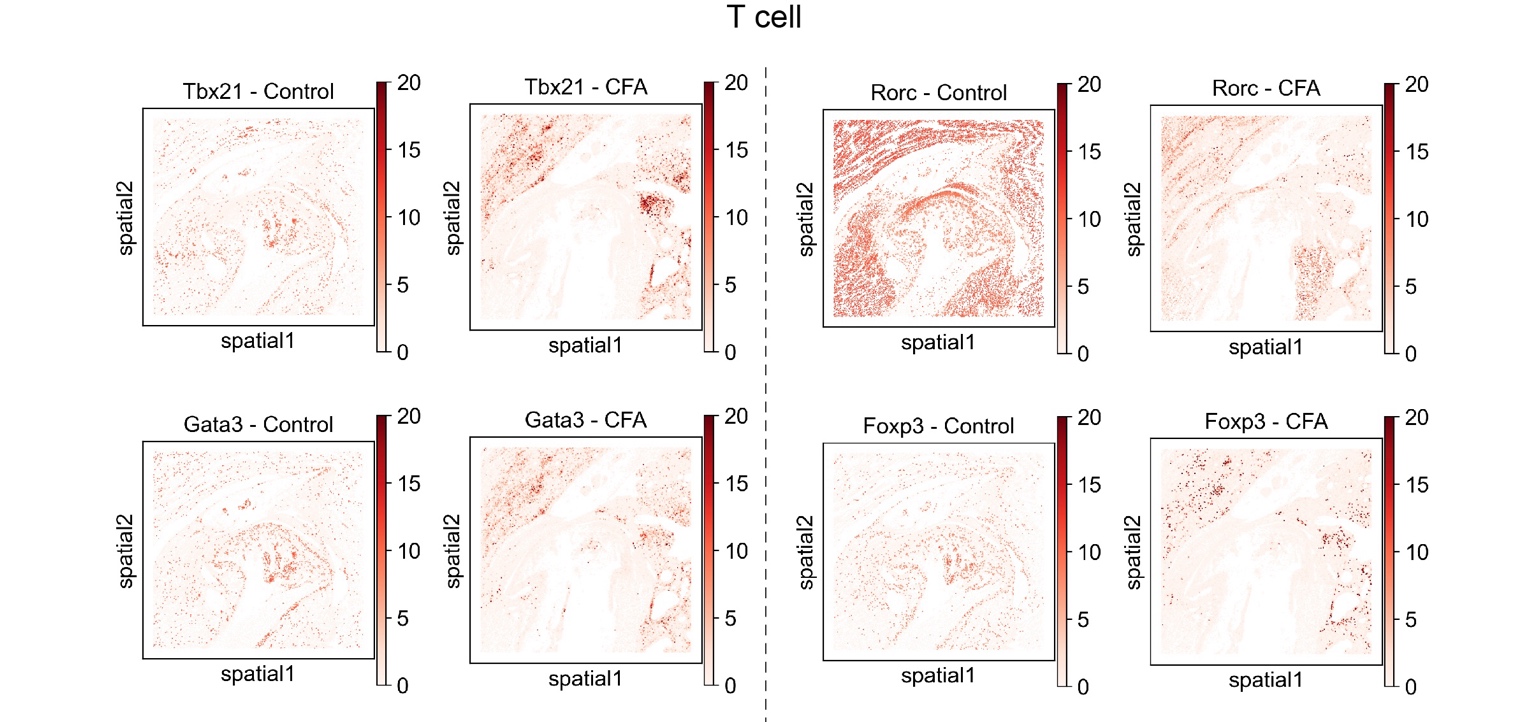

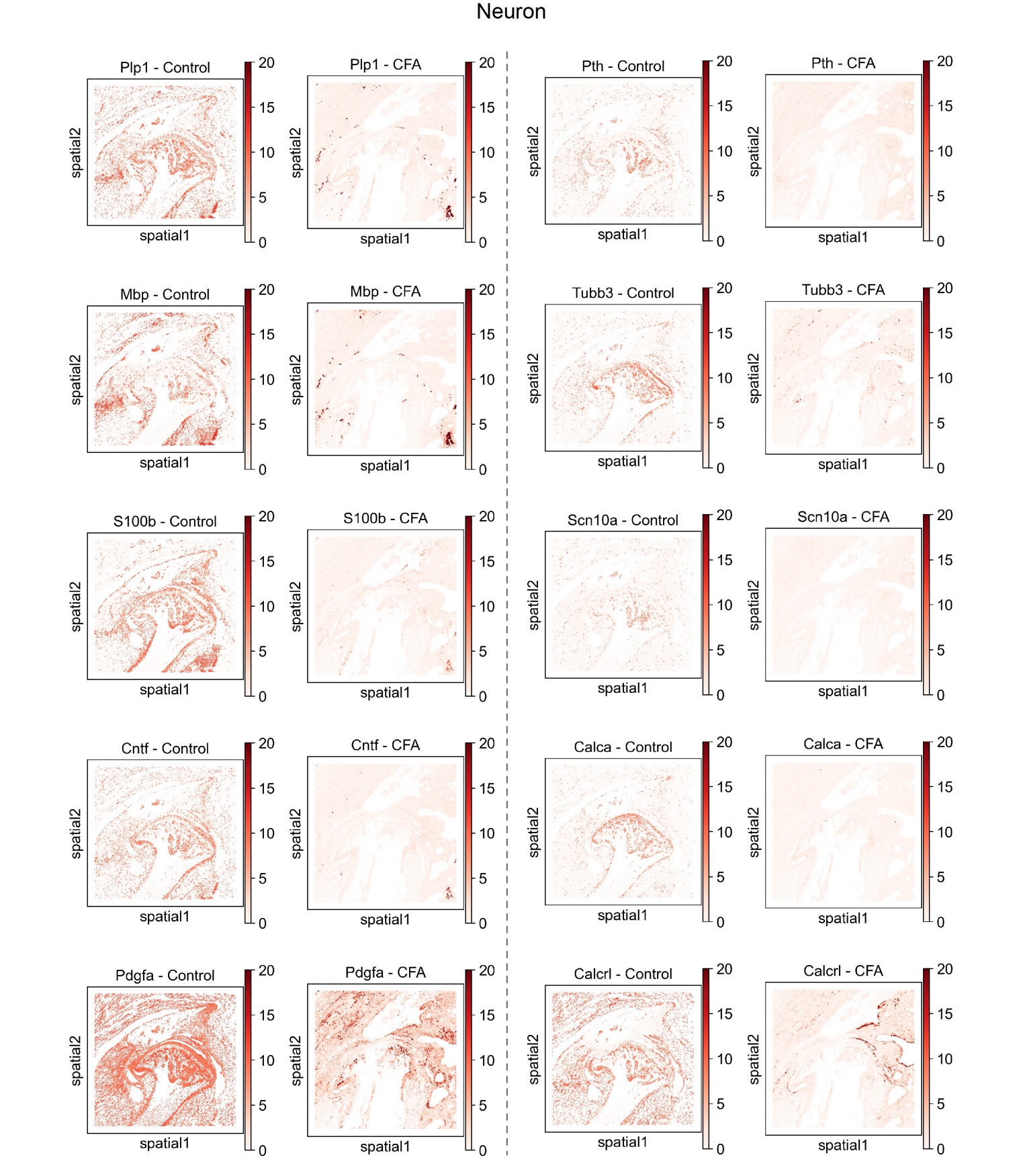

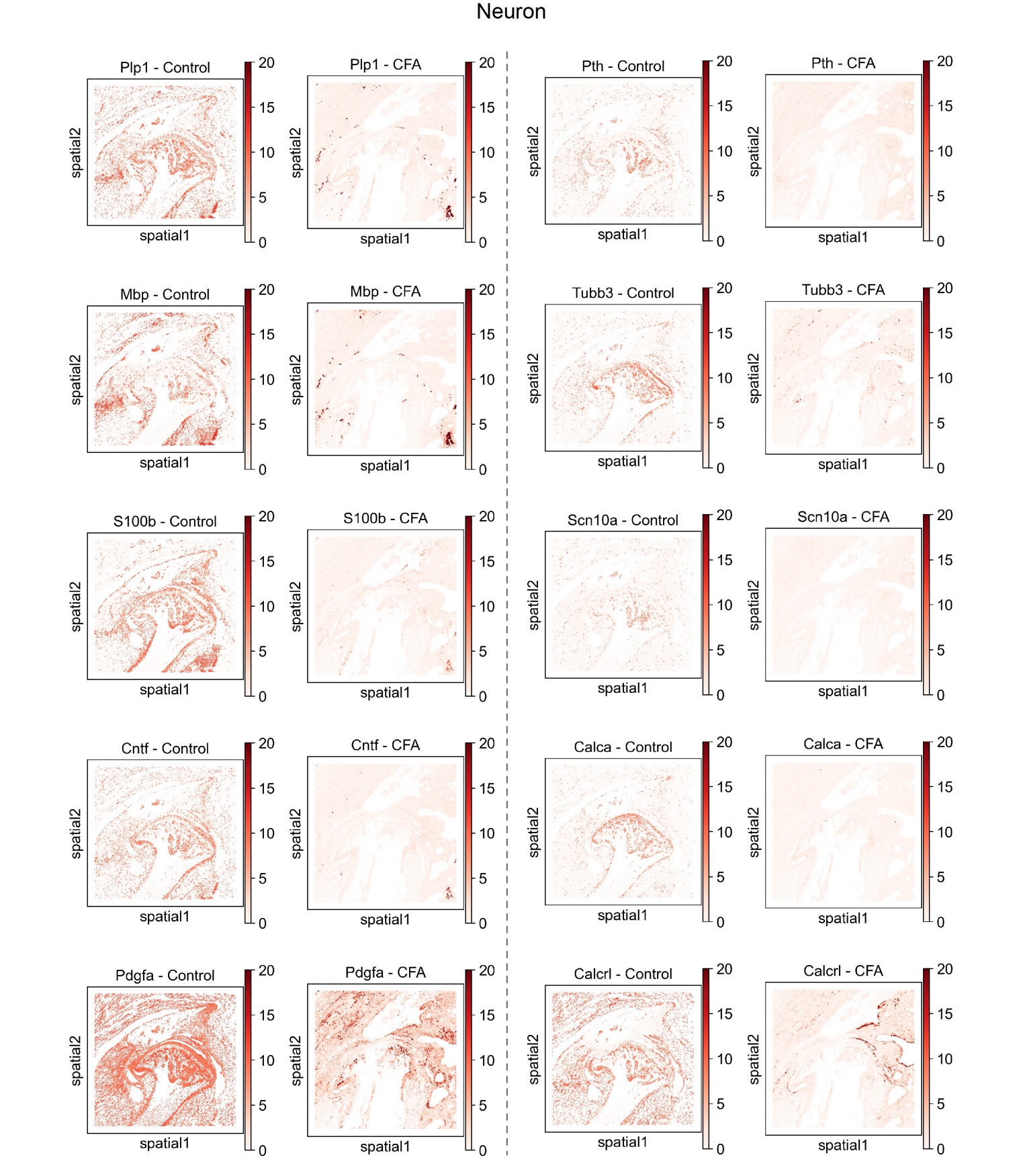

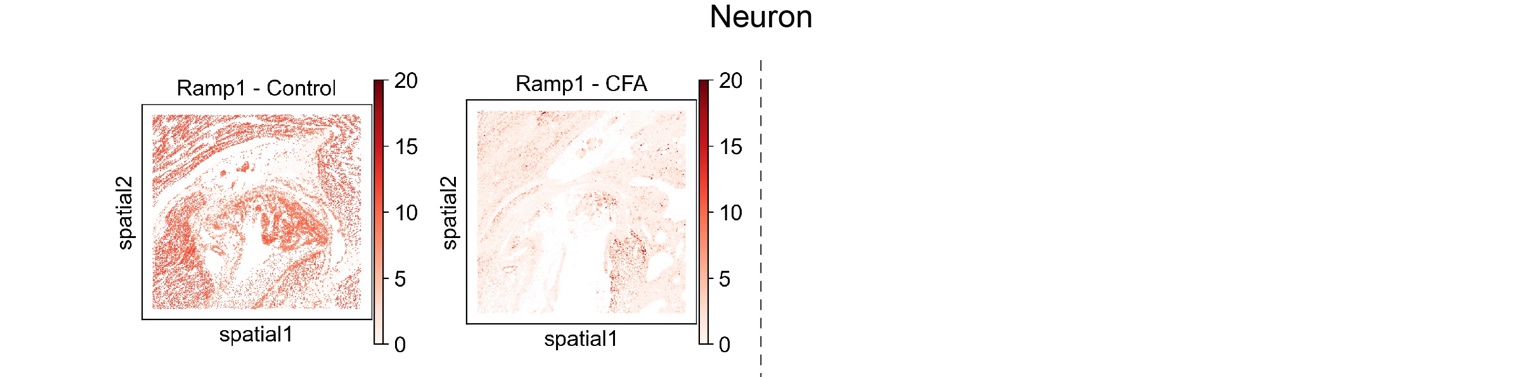

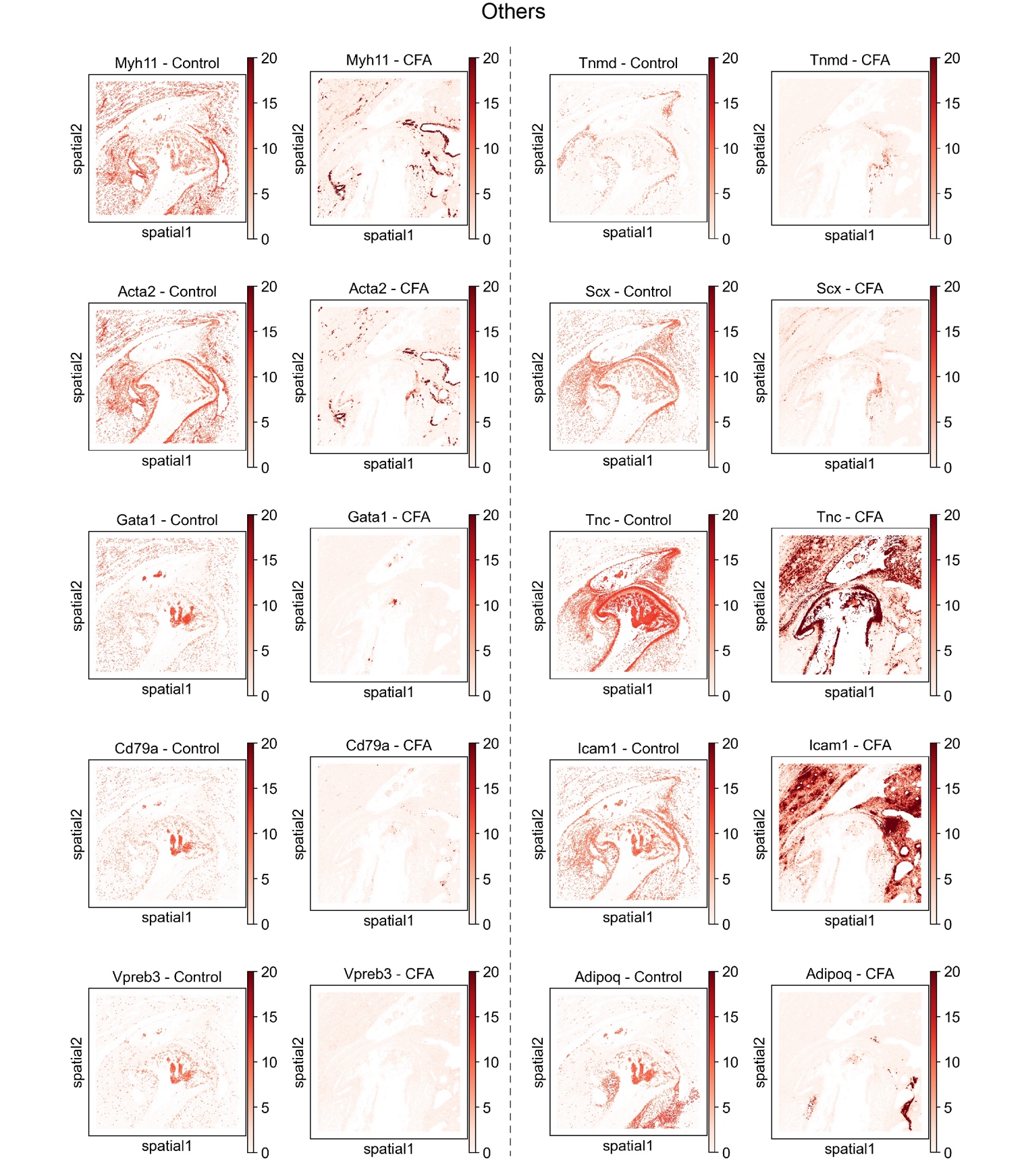
**

**
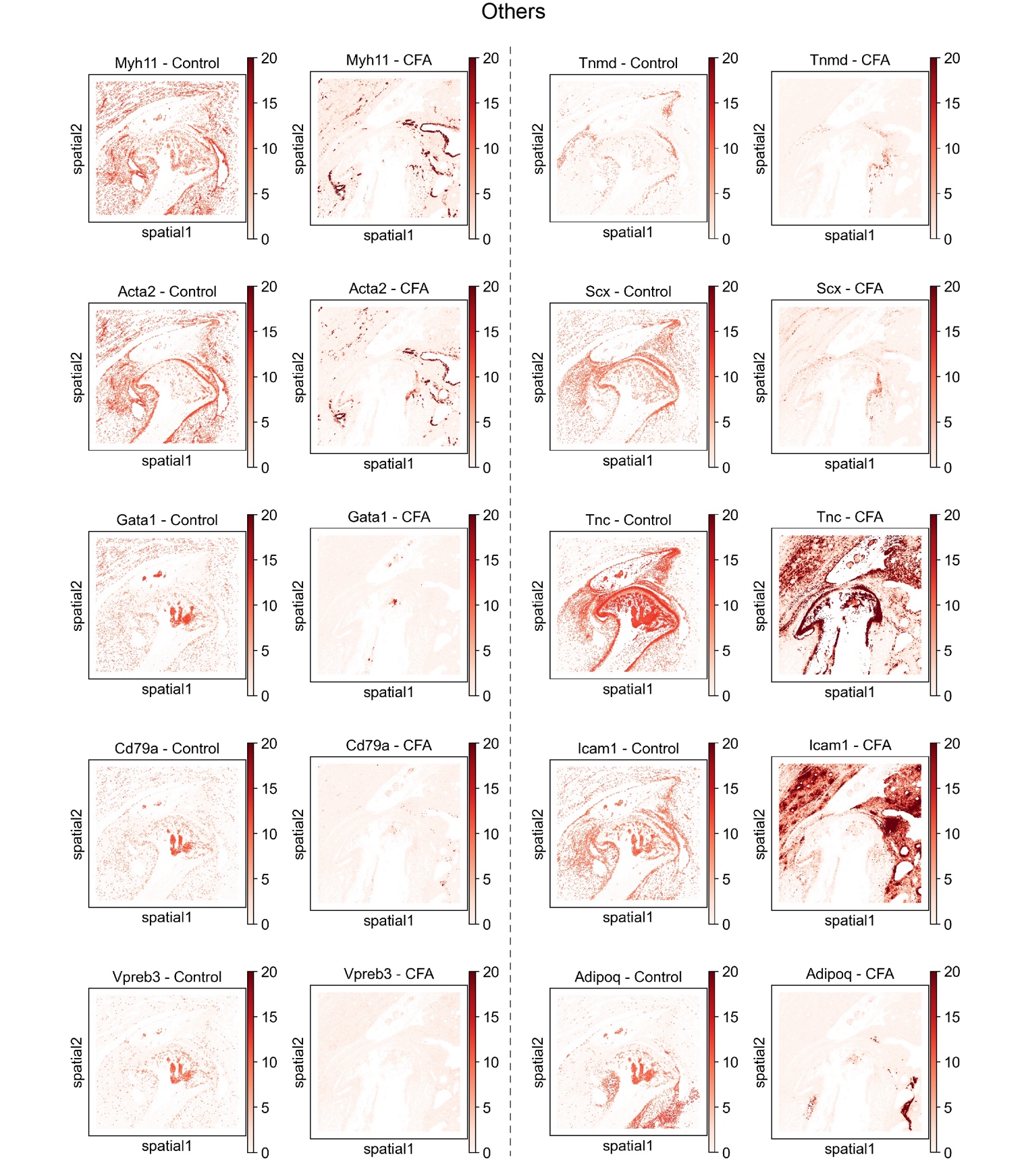

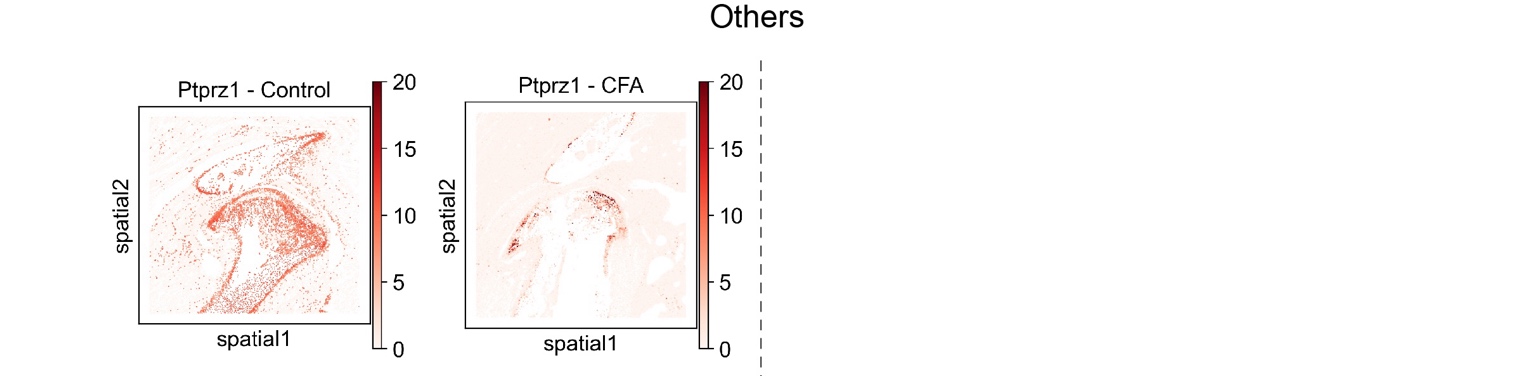
**

**Figure S1. Spatial expression pattern of genes of interest,** related to **Figure 1.** Spatial expression of individual genes in PBS- or CFA-treated mouse TMJ samples were plotted by Scanpy. Expression level is normalized from 0 (white) to 20 (dark red).


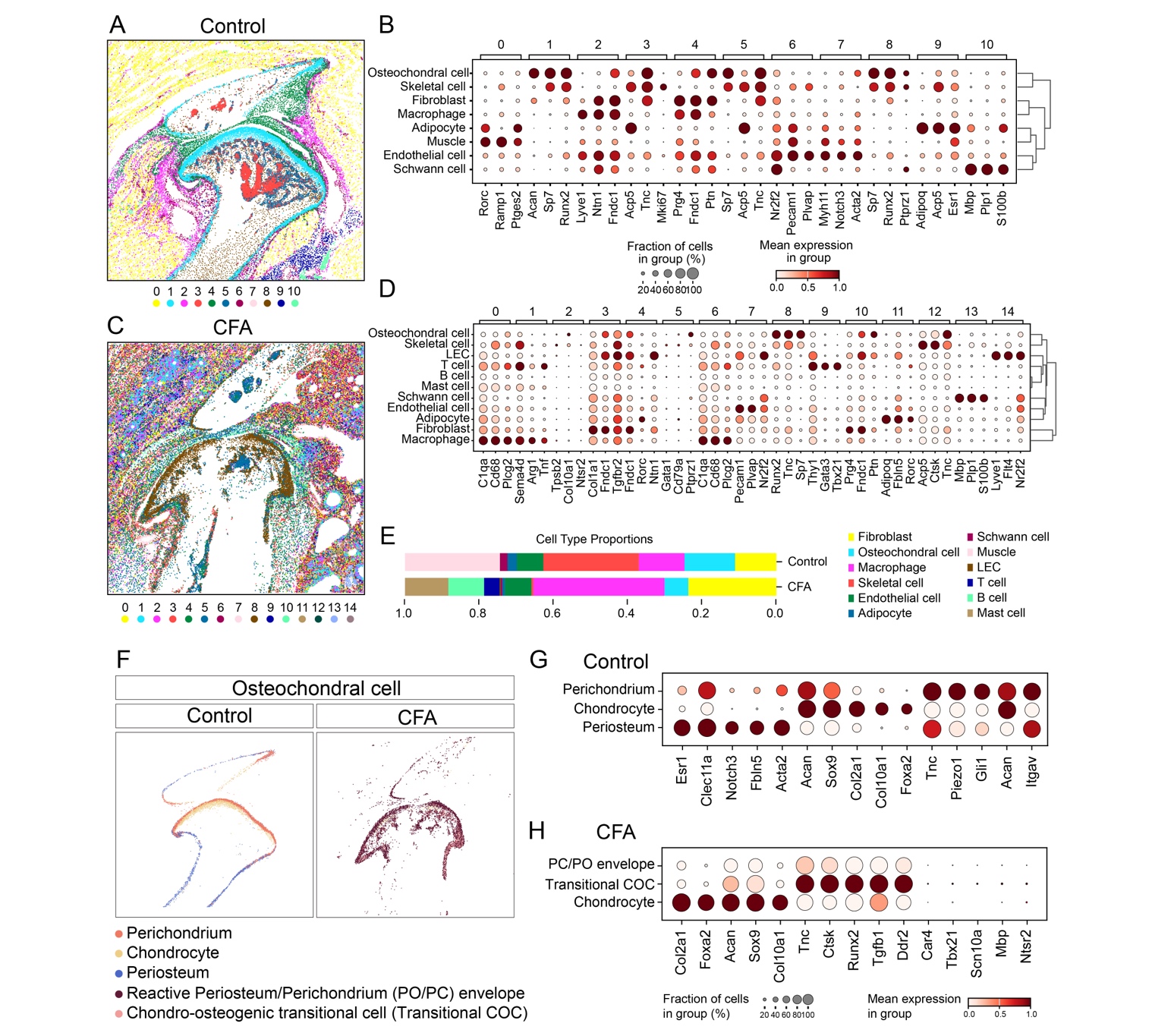
**Figure S2**. **TMJ cell type annotation analysis** **of Control and CFA conditions**, related to **Figure 1**. (A) Spatial plot of the Control sample. (B) Dot plot of differentially expressed genes in Control. (C) Spatial plot of the CFA sample. (D) Dot plot of differentially expressed genes in CFA condition. (E) Cell type proportion analysis after assigning cell types. (F) seqFISH figures analyzed by Scanpy showing location of the osteochondral subpopulations. Different colors labeled different cell types. (G-H) Dot plot of top-ranked gene expression in osteochondral cell subpopulations in control and CFA-induced arthritic TMJ.

**
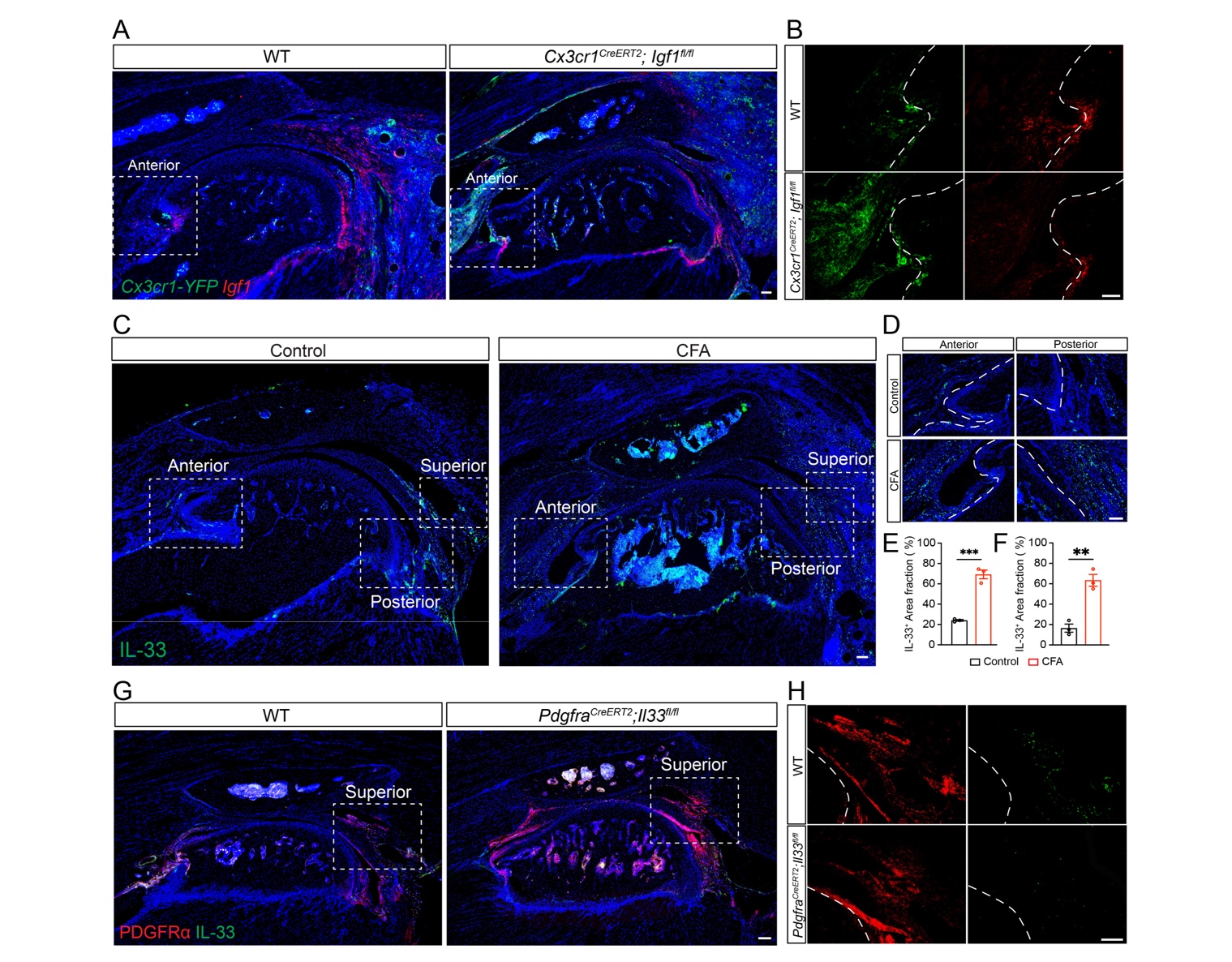
Figure S3**. **Validation of *Igf1* deletion in macrophages and IL-33 in fibroblasts,** related to **Figure 4 and 6**. (A) RNAscope and Immunofluorescence staining of Igf1 (red) and Cx3cr1-YFP (green) in the anterior region of the TMJ. DAPI stains nuclei (blue). Images in B (20× objective) are enlargements of boxed regions of TMJ in A (4× objective) at the anterior regions. Scale bar: 100 μm. (C-D) Immunofluorescence staining of IL-33 (green) in different regions surrounding the TMJ. DAPI stains nuclei (blue). Images in B (20× objective) are enlargements of boxed regions of TMJ in A (4× objective) at the anterior and posterior positions. Scale bar: 100 μm. (E-F) Quantification of the IL-33^+^ area fraction. (G) Immunofluorescence staining of IL-33 (green) and PDGFRα (red) in the superior region of the TMJ. DAPI stains nuclei (blue). Images in H (20× objective) are enlargements of boxed regions of TMJ in G (4× objective) at the superior regions. Scale bar: 100 μm. All data are represented as mean ± SEM calculated by Student’s *t*-test, n = 3, ***p*<0.01, ****p*<0.001.
